# eSPRESSO: topological clustering of single-cell transcriptomics data to reveal informative genes for spatio–temporal architectures of cells

**DOI:** 10.1186/s12859-023-05355-4

**Published:** 2023-06-15

**Authors:** Tomoya Mori, Toshiro Takase, Kuan-Chun Lan, Junko Yamane, Cantas Alev, Azuma Kimura, Kenji Osafune, Jun K. Yamashita, Tatsuya Akutsu, Hiroaki Kitano, Wataru Fujibuchi

**Affiliations:** 1 Bioinformatics Center, Institute for Chemical Research, Kyoto University, Gokasho, Uji, Kyoto 611-0011 Japan; 2 Life Sciences, IBM Consulting, IBM Japan Ltd., 19-21 Nihonbashi Hakozaki-cho , Chuo-ku, Tokyo 103-8510 Japan; 3 Center for iPS Cell Research and Application (CiRA), Kyoto University, 53 Kawahara-cho, Sho-goin, Sakyo-ku, Kyoto, 606-8507 Japan; 4 Institute for the Advanced Study of Human Biology (ASHBi), Kyoto University, Yoshida-Konoe-cho, Sakyo-ku, Kyoto, 606-8501 Japan; 5 The Systems Biology Institute, Tokyo, Japan; 6 Okinawa Institute of Science and Technology Graduate School, Okinawa, Japan; 7 Sony Computer Science Laboratories, Inc., Tokyo, Japan; 8 Sony AI, Inc., Tokyo, Japan; 9 The Alan Turing Institute, London, UK

**Keywords:** Spatio–temporal tissue reconstruction, Cellular organization, Spatial discriminator gene, Self-organizing map clustering, Markov chain Monte Carlo optimization, Developmental trajectory

## Abstract

**Background:**

Bioinformatics capability to analyze spatio–temporal dynamics of gene expression is essential in understanding animal development. Animal cells are spatially organized as functional tissues where cellular gene expression data contain information that governs morphogenesis during the developmental process. Although several computational tissue reconstruction methods using transcriptomics data have been proposed, those methods have been ineffective in arranging cells in their correct positions in tissues or organs unless spatial information is explicitly provided.

**Results:**

This study demonstrates stochastic self-organizing map clustering with Markov chain Monte Carlo calculations for optimizing informative genes effectively reconstruct any spatio–temporal topology of cells from their transcriptome profiles with only a coarse topological guideline. The method, eSPRESSO (enhanced SPatial REconstruction by Stochastic Self-Organizing Map), provides a powerful in silico spatio–temporal tissue reconstruction capability, as confirmed by using human embryonic heart and mouse embryo, brain, embryonic heart, and liver lobule with generally high reproducibility (average max. accuracy = 92.0%), while revealing topologically informative genes, or spatial discriminator genes. Furthermore, eSPRESSO was used for temporal analysis of human pancreatic organoids to infer rational developmental trajectories with several candidate ‘temporal’ discriminator genes responsible for various cell type differentiations.

**Conclusions:**

eSPRESSO provides a novel strategy for analyzing mechanisms underlying the spatio–temporal formation of cellular organizations.

**Supplementary Information:**

The online version contains supplementary material available at 10.1186/s12859-023-05355-4.

## Background

Analysis of biological functions and disease mechanisms based on high-throughput single-cell RNA-sequencing (scRNA-seq) is becoming a widely accepted and fundamental technique in cell biology [1–3]. In particular, methods for high-throughput, spatially resolved scRNA-seq have been developed and are attracting attention as novel analytics in this field. Single-molecule fluorescence in situ hybridization (FISH) [4] has been widely used for quantitating transcript numbers at single-cell resolution while preserving 3D locations of cells, often within the context of a diseased tissue of interest. Highly multiplexed methods such as seqFISH [5] or MERFISH [6] have been employed to measure the transcripts for over 10,000 genes of target cells in 3D locations. High-resolution, 2D-grid primer-based RNA sequencing of tissues fixed on a glass plate has also been developed for mapping transcript abundance and drawing spatial cellular location maps after reconstructing 3D images using multiple 2D maps [7]. However, these methods are still in their infancy and require further improvement in terms of practical costs and convenience for whole-organ research.

Alternatively, several computational methods for reconstructing 3D tissues by estimating the spatial positions of individual cells using gene expression data obtained by scRNA-seq have been reported [8–14]. These methods can be roughly divided into two approaches: the landmark approach and the ab initio approach. The landmark approach estimates the 3D position of each cell on the basis of gene expression profiles while using the spatial information of marker genes obtained by other experiments such as in situ hybridization [8–10]. Conversely, the ab initio approach assigns each cell to a 3D space according to the principal component score calculated from gene expression profiles without using spatial reference information [11–14]. Although current principal component analysis (PCA)-based methods may be insufficient for 3D reconstruction, an ab initio approach that does not depend on the spatial information of marker genes is promising and desired for 3D reconstruction. Other methods for the reconstruction of spatial relationships of cells from non-spatial scRNA-seq data have been developed, including novoSpaRc [15], SpaOTsc [16], ScoMap [17], and CSOmap [18], which have provided new biological insights into spatial gene expression patterns and spatially informative genes within tissue. However, although these methods are able to project cell data to a 2D or 3D pseudo space or reference map, the reproducibility of real tissue structure is not thoroughly discussed. Advanced experimental techniques for the simultaneous acquisition of gene expression profiles and cell locations have also been developed and become widely used, such as 10x Visium, Slide-seq [19, 20], HDST [21], and Stereo-seq [22]. Accordingly, several spatial transcriptome analysis methods that use the output of the aforementioned techniques have been proposed, including SpaGCN [23], Squidpy [24], and Spatial-ID [25]. However, most of them are aimed at the segmentation of tissue and the detection of spatially informative genes, not at reconstruction.

Previously, we reported a novel 3D reconstruction method using SPRESSO (SPatial REconstruction by Stochastic Self-Organizing Map) [26], which features gene selections based on gene sets from Gene Ontology (GO) [27, 28]. The method yielded high success rates of 3D reconstructions of mouse gastrula stage embryos and demonstrated a remarkable ability to identify spatial discriminator genes (SDGs) that contribute to differentiation and tissue morphogenesis. This method, however, was preliminary and simply projected four domains of mouse gastrula to only a cubic structure, and thus was inapplicable to more complicated tissue structures of organs such as the heart or pancreas.

In this work, we remove the limitations of structural presentations by introducing graph-based self-organizing map (SOM) clustering for the reconstruction of any topology of cell domains in tissues, as long as they can be drawn as network diagrams or graphs. The basic concept of the graph-based SOM clustering was reported in 1990 by Kohonen et al. [29] who introduced a Kohonen map in 1982 [30] as an artificial neural network and a computationally convenient abstraction building on the biological models of neuronal systems [31] or morphogenesis models by Turing [32]. Many useful topological structures other than square grids, such as hexagonal grids, toroidal grids whose opposite ends are seamlessly connected [33], have been introduced. However, graph-network representation is the simplest and most abstract yet comprehensive method for describing the relationships between cell domains in tissues. Here, we applied graph-based SOM clustering to various types of mouse and human organs to reconstruct or infer cellular organizations while revealing informative SDGs that can be ranked using novel virtual knockout (VKO) analysis. Furthermore, we extended graph-based SOM clustering to temporal analysis to elucidate the cell lineage trajectories of human pancreatic organoids. We used uniform manifold approximation and projection (UMAP) [34] to visually confirm topologies and draw new insights from the resultant topologies of cell domains.

## Results

All the datasets used in this paper are summarized in Table 1.

**Table 1:** Datasets and performances of 3D structure reconstructions (score = accuracy + ARI)

Data name	References	#Domains	#Cells	#Initial genes	#Consensus SDGs (≥ 3) of max scores	Average of max scores	Average max accuracy	Average max ARI
Mouse embryo (E7.0)	Peng, Guangdun, et al. [57]	4	41 (sections)	129	87	2.00	1.00	1.00
Mouse embryo (E7.5)	Peng, Guangdun, et al. [36]	7	83 (sections)	295	36	1.93	0.98	0.95
Mouse brain (ALM)	Tasic, Bosiljka, et al. [39]	3	3809	1363	61	2.00	1.00	1.00
Mouse brain (VISp)		4	7049	2377	10	2.00	1.00	1.00
Mouse heart (E7.75)	de Soysa, T. Yvanka, et al. [41]	4	1259	908	19	1.99	1.00	0.99
Mouse heart (E8.25)		7	3331	1609	39	1.86	0.96	0.90
Mouse heart (E9.25)		7	3911	1699	20	1.79	0.94	0.85
Mouse liver	Halpern, Keren Bahar, et al. [10]	9	1415	223	66	1.30	0.82	0.48
Human heart (PCW 4.5–5)	Asp, Michaela, et al. [40]	8	238 (spots)	64	43	1.23	0.75	0.48
Human heart (PCW 6.5)		8	1515 (spots)	529	77	1.40	0.85	0.56
Human heart (PCW 9)		7	1358 (spots)	193	70	1.50	0.86	0.64
Human pancreas organoid (S3-S6)	Veres, Adrian, et al. [47]	18	20,205	469	83	1.15	0.76	0.39

### Topological clustering of gene expression data by graph-based SOM with gene set optimization

**Fig. 1:**
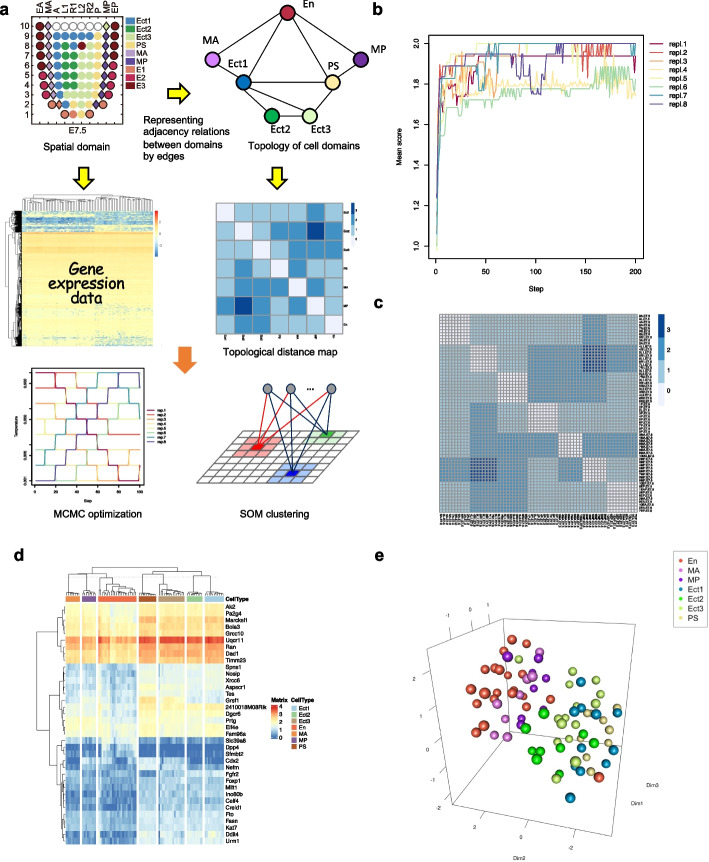
eSPRESSO analysis for graph-based SOM clustering to detect spatial discriminator genes (SDGs). **a** Schema of the algorithm. eSPRESSO performs SOM clustering using MCMC algorithm with gene expression data based on graph representation of topology among cell or tissue types. A, anterior; P, posterior; L1, anterior left lateral; R1, anterior right lateral; L2, posterior left lateral; R2, posterior right lateral; MA, anterior mesoderm; MP, posterior mesoderm; EA, anterior endoderm, EP, posterior endoderm; Ect1–3, ectoderm; PS, primitive streak; E1–3 (En), endoderm; rep.1–8, replica. **b** Optimization of SDGs by replica exchange to increase cell or tissue type clustering accuracy while preserving topological consistency defined by weighted ARI + accuracy score. **c** A topological distance map of cells or tissues for the original (lower left) and resultant (upper right) clusters. **d** Gene expression heatmap for the optimized SDGs. **e** 3D reconstruction of cell or tissue types with SDGs using UMAP

Inspired by the original Kohonen SOM learning theory [30], we extended our previously developed method, SPRESSO [26], to graph-based eSPRESSO (enhanced-SPRESSO) topological clustering, which is theoretically applicable to any cell-to-cell relationships of tissues or organs if they are represented as a graph structure. The basic algorithm for topological clustering of cells is a combinatorial optimization to find the best gene sets to reproduce known structures, or a topological guidelines for learning the gene expression vectors of cells, as schematically shown in Fig. 1a. Given the original topology of cell domains, we can calculate the accuracy of topology reproducibility by counting the correct and incorrect edges between cells, either connected or unconnected, after a learning process. We can also calculate the clustering performance without considering the topology of the cell domains using the adjusted Rand index (ARI). As an integrated score of these two metrics, we calculate the clustering score defined by the weighted sum of the accuracy and ARI, where we set equal weights. We used the Markov chain Monte Carlo (MCMC) [35] method to optimize the best gene sets to attain the maximum topological clustering score. The evaluation and optimization processes require a known topological structure, which makes the method not fully unsupervised. However, the purpose of eSPRESSO is not simply to search for marker genes of known domains, but also to search for the best gene sets that reconstruct the adjacency relationship between the domains. Therefore, graph-based SOM learning considers tissue topology to detect spatially contributing genes showing expression gradients across neighboring cell domain networks. We previously tuned the SOM learning performance by introducing a stochastic-learning version of SOM, or stochastic-SOM [26], which enhances learning efficiency in the later phase where the extent of learning ability usually decreases monotonically. In preliminary implementations with mouse gastrula embryo (E7.0), which use the same dataset as that in SPRESSO [26], eSPRESSO showed perfect reproducibility, i.e., the average of the maximum scores of ten runs was 2.00 (accuracy: 1.00 + ARI: 1.00) (Table 1). Furthermore, the 87 consensus SDGs that were found three or more times in the ten runs suggest biologically interesting genes that contribute to embryonic development, such as “cell adhesion”, “cell differentiation”, or “nervous system development”, by GO enrichment analysis (Additional file 1: Fig. S1).

We first tested the ability of eSPRESSO with mouse E7.5 gastrula embryo [36], whose topological structures are too complicated to be handled or reproduced by our previous SPRESSO, by generating a graph structure of seven cell domains (Ect1, Ect2, Ect3, PS, MA, MP, E1-E2-E3) derived from domain-to-domain contact relationships in the original paper (Fig. 1a). We optimized the gene set to maximize topology reproducibility by 1000 steps of MCMC calculations. To enhance the efficient optimization and stable reproducibility, we performed replica exchange [37] based on eight parallel MCMC processes (Fig. 1b). In this study, we conducted ten runs of clustering, each of which randomly downsampled ten (or all if only fewer cells existed) cells from each of the domains, to calculate scores. We used gene expression data from 64 sections. The average of the maximum scores of ten runs was 1.93 (accuracy: 0.98 + ARI: 0.95) after the MCMC optimization, and the topological distance map which shows the shortest path distances of samples for the original (lower triangle) and reconstructed (upper triangle) structures is shown (Fig. 1c). A heatmap of gene expression data for the optimized 36 consensus SDGs that were found three or more times in the ten runs is also shown (Fig. 1d). The final topological clustering result for the 83 sections using SDGs was confirmed visually by UMAP (Fig. 1e and Additional file 2: Movie S1).

### Reconstruction of simple layer and complicated structures by eSPRESSO

**Fig. 2:**
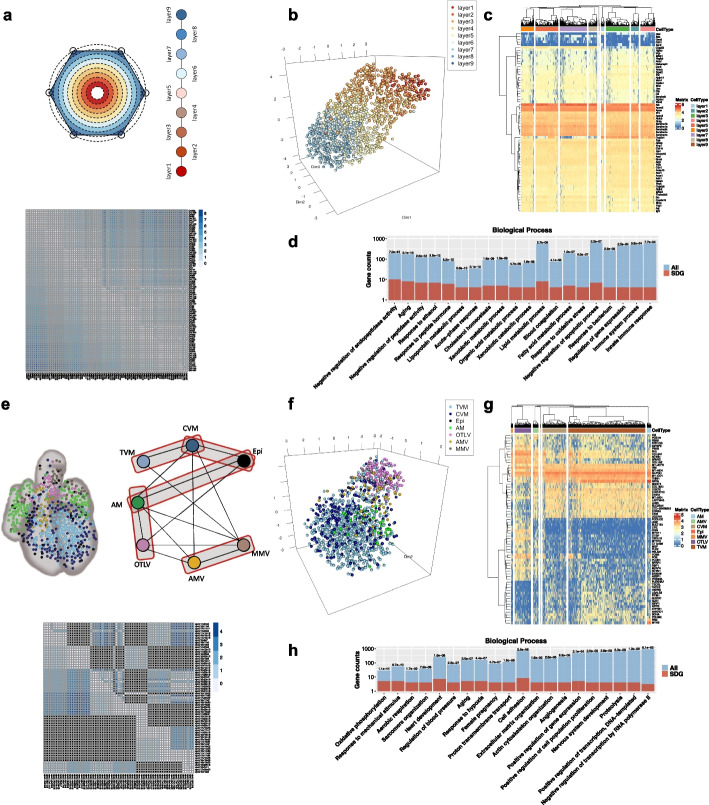
Examples of eSPRESSO analysis in mouse liver lobule **a**–**d** and PCW 9 human developmental heart **e**–**h**. **a, e** Graph representation of topology of cells or tissues and a topological distance map of cells for the original (lower left) and resultant (upper right) clusters. We adopted sparse network topology for human developmental heart because of uncertainty. **b, f** 3D reconstruction of cells with SDGs using UMAP. **c, g** Gene expression heatmap for the optimized SDGs. **d, h** GO mapping of SDGs in Biological Process. The *p*-values determined by hypergeometric distribution statistics are shown on top of bars

To evaluate the performance of eSPRESSO, we applied the method to a one-dimensional layer and complicated topological structures of the cell domains. We first tried to reconstruct mouse liver lobule structure data, where nine layers of cell domains or tissues from a concentric circle exist, as provided by Halpern et al. [10] (Fig. 2a–d). eSPRESSO reconstructed the relationships of 90 cells that are randomly selected from each of the nine domains, and the average of the maximum scores of ten runs was 1.30 (accuracy: 0.82 + ARI: 0.48). The 66 resultant consensus SDGs that were found three or more times in the ten runs represent many known liver-specific genes such as cytochrome P450 (*Cyp2f2*, *Cyp2e1*, *Cyp2a12*, and *Cyp2d9*), fibrinogen alpha chain (*Fga*), and hepatocyte growth factor activator (*Hgfac*), and GO enrichment analysis provided the candidates for layer-dependent functions of the liver lobules (Fig. 2c, d and Additional file 3: Table S1). Ten differentially expressed genes, *Cdh1*, *Cyp2e1*, *Cyp2f2*, *Gas2*, *Glul*, *Gst3*, *Npr2*, *Pck1*, *Por*, and *Sds*, between periportal and perivenous hepatocytes previously reported by Braeuning et al*.* [38] overlap with our consensus SDGs but many others are also found in eSPRESSO results. This was probably due to the difference between the differentially expressed genes in the two hepatocyte regions in their study and the gradiently expressed genes across the nine layers in our topological clustering. Indeed, the visual projection by UMAP clustering of all 1415 cells using the consensus SDGs indicated that there were clear layer structures although it did not reproduce the circular structure with this layer topology (Fig. 2b and Additional file 4: Movie S2). We also tested the topology reproducibility of the layer structures using scRNA-seq data from two areas in the mouse brain neocortex: ALM (anterior lateral motor cortex) and VISp (primary visual cortex), which have three and four layers, respectively [39]. Both showed perfect reproducibility, namely, the average of the maximum scores of ten runs was 2.00 (accuracy: 1.00 + ARI: 1.00) (Table 1).

Next, we attempted to reconstruct complicated topological structures using eSPRESSO. As an example of organs with complicated tissue structures, we used spatial transcriptomics data of the human developmental heart [40]. We first reconstructed eight domain (TVM, CVM, Epi, CBIC, MMV, AMV, OTLV, and AM) structures for embryonic heart at postconceptional week (PCW) 4.5–5 or 6, and seven domain (the same domains as above except CBIC) structures for that at PCW 9 with the average of the maximum scores of ten runs being 1.23–1.50 (accuracy: 0.75–0.86 and ARI: 0.48–0.64) (Table 1). For PCW 9 embryonic heart analysis, we prepared two topological guidelines, dense and sparse network models (Additional file 1: Fig. S2). All of the seven domains were connected either directly or indirectly in the dense network, whereas two separated domain clusters (TVM–CVM–Epi–AM–OTLV and AMV–MMV) were generated in the sparse network (enclosed by red rectangles in Fig. 2e). Because of the uncertainty of the complicated domain relationships in the dense network model, we adopted the sparse network model for eSPRESSO for the 3D reconstruction of the embryonic heart structure. Surprisingly, the resultant UMAP view of all 1358 spots suggests that the two separated domains should be connected, which resembles the original PCW 9 embryonic heart data [40], where the small domain cluster (AMV–MMV) is appropriately located between the OTLV and the other domains (Fig. 2e, f and Additional file 4: Movie S2). This result indicates that eSPRESSO may be useful for reconstructing the spatial relationships of cells, even with limited topological information. Furthermore, the 70 consensus SDGs that were found three or more times in ten runs may suggest biologically interesting genes that contribute to the self-organization of the embryonic heart, such as “heart development” or “cell adhesion”, by GO enrichment analysis (Fig. 2g, h and Additional file 5: Table S2).

### Application to virtual knockout experiment

**Fig. 3:**
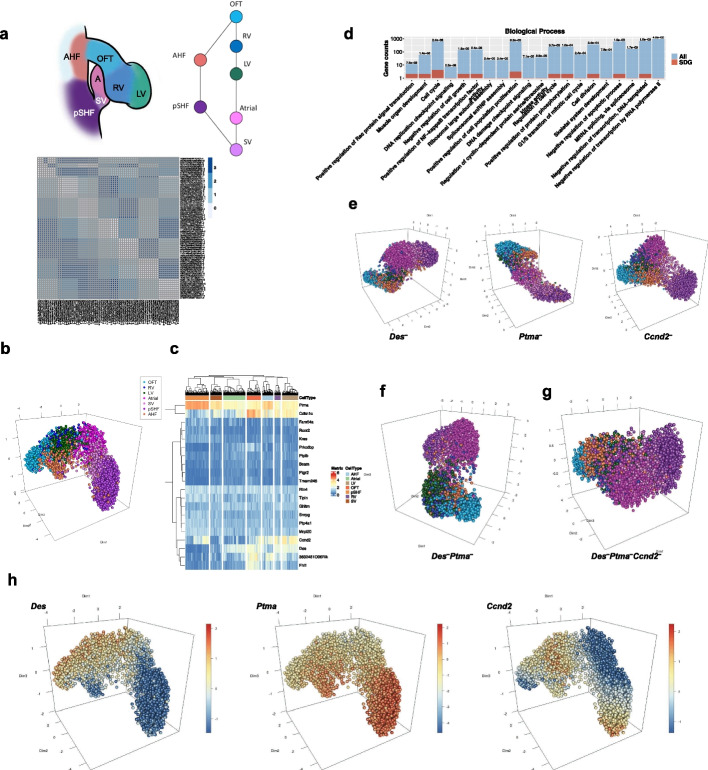
Virtual knockout (VKO) analysis for mouse developmental heart in eSPRESSO. **a** Graph representation of topology of cells or tissues and a topological distance map of cells for the original (lower left) and resultant (upper right) clusters. **b** 3D reconstruction of cells with SDGs using UMAP. **c** Gene expression heatmap for the optimized SDGs. **d** GO mapping of SDGs in Biological Process. The *p*-values determined by hypergeometric distribution statistics are shown on top of bars. **e–g** 3D reconstruction of cells with VKO analysis by **e** single gene KO: *Des*^–^ (left), *Ptma*^–^ (center), or *Ccnd2*^–^ (right); **f** double gene KO: *Des*^–^*Ptma*^–^; and **g** triple gene KO: *Des*^–^*Ptma*^–^*Ccnd2*^–^. **h** Gene expression gradients in reconstructed 3D structure for VKO genes

One of the most promising applications of eSPRESSO is in silico VKO experiments because real knockout experiments in human or animal studies may result in lethality or evoke ethical problems. The development of the mouse heart is characterized by the spatially and temporally controlled expansion and differentiation of cardiogenic progenitor cells into an initially single primitive heart tube, from which the complex organ structure of the heart arises. We used scRNA-seq data of mouse embryonic heart (E9.25) [41], which represent the pseudo-circular structure of seven domains, of which five project from the anterior and posterior second heart fields (AHF and pSHF) (Fig. 3a). We performed ten runs of eSPRESSO clustering and reconstructed the topology with a high average maximum score of 1.79 (accuracy: 0.94 + ARI: 0.85). (Table 1). The visual projection by UMAP clustering of all 3331 cells using the 20 consensus SDGs with the same frequency as above shows a circular representation of tissue domains, which is consistent with the original structure (Fig. 3b and Additional file 6: Movie S3). The consensus SDGs include novel as well as known genes in mouse heart development or cardiac diseases, such as PICALM interacting mitotic regulator (*Fam64a*) [42], four and a half LIM domain protein 1 (*Fhl1*) [43], and others (Fig. 3c, d, Table 2 and Additional file 7: Table S3).

**Table 2:** Virtual knockout genes and performances of 3D structure reconstructions by running topological clustering 100 times (score = accuracy + ARI)

VKO gene	Frequency	Max score	Max accuracy	Max ARI	Mean score	Mean accuracy	Mean ARI
noKO	NA	1.470	0.828	0.657	1.143	0.721	0.422
Fam64a	3	1.55	0.86	0.70	1.15	0.72	0.43
Kras	3	1.49	0.83	0.66	1.15	0.72	0.42
Ptp4a1	3	1.49	0.83	0.66	1.15	0.72	0.42
Ghitm	5	1.49	0.83	0.66	1.14	0.72	0.42
Bcam	3	1.49	0.83	0.66	1.14	0.72	0.42
Ptplb	3	1.49	0.83	0.67	1.14	0.72	0.42
Tmem246	4	1.48	0.82	0.66	1.14	0.72	0.42
Snrpg	3	1.44	0.81	0.63	1.14	0.72	0.42
Rtn4	3	1.49	0.83	0.66	1.14	0.72	0.42
Tipin	3	1.57	0.86	0.71	1.14	0.72	0.42
Ptgr2	4	1.49	0.83	0.66	1.14	0.72	0.42
Rcor2	3	1.47	0.82	0.66	1.14	0.72	0.42
Mrpl20	3	1.47	0.82	0.65	1.14	0.72	0.42
Prkcdbp	3	1.44	0.81	0.65	1.13	0.72	0.42
Fhl1	4	1.49	0.83	0.66	1.13	0.72	0.41
Cdkn1c	5	1.36	0.81	0.60	1.12	0.72	0.41
3632451O06Rik	4	1.53	0.84	0.69	1.11	0.71	0.40
Ccnd2	5	1.41	0.80	0.62	1.08	0.71	0.37
Ptma	3	1.36	0.79	0.60	1.07	0.70	0.37
Des	3	1.39	0.80	0.60	1.05	0.69	0.36
Des:Ptma	3:3	1.22	0.76	0.46	0.92	0.66	0.25
Des:Ptma:Ccnd2	3:3:5	1.24	0.77	0.49	0.97	0.67	0.30

To understand the topological importance of SDGs, we performed VKO experiments with eSPRESSO topological reconstruction by deleting genes one by one. For each VKO, we ran topological clustering 100 times with different random seeds and calculated the mean score (Table 2). Among the 20 SDGs, desmin (*Des*), prothymosin alpha (*Ptma*), and cyclin D2 (*Ccnd2*) are the most influential genes for mouse heart structure, as indicated in the literature [44–46], which decreases the normal mean score of 1.143 to 1.05, 1.07, and 1.08, respectively. The visual inspection by UMAP clustering for individual VKO experiments (*Des*^−^, *Ptma*^−^, or *Ccnd2*^−^) reveals that the reconstructed topologies had lower domain clustering resolutions with the same plotting parameters as normal SDGs (Fig. 3e and Additional file 6: Movie S3). We then performed multiple VKO experiments by deleting *Des* and *Ptma*, which dramatically decreased the mean score to 0.92. We further performed multiple VKO experiments by deleting all three genes, *Des*, *Ptma*, and *Ccnd2*, but the mean score did not decrease further; rather, it slightly increased to 0.97 (Table 2). The UMAP by multiple VKO experiments was indeed aberrant compared with that of normal SDGs, where the domains become unclear and fused (Fig. 3f, g and Additional file 6: Movie S3), indicating that the three genes may be vital to cardiac development. Indeed, the gene expression distribution of the three genes clearly and spatially indicates that the genes are reversely and complementarily up-regulated across cardiac regions; *Des* and *Ptma* are mainly up-regulated in the OFT and pSHF domains, respectively, and down-regulated vice versa, whereas *Ccnd2* is mainly up-regulated in the LV and the pSHF domains but down-regulated in the middle of both, that is, in the Atrial domain (Fig. 3h and Additional file 8: Movie S4). This tendency of the gene expression gradient was also confirmed in the reconstructed model of mouse developmental heart by novoSpaRc [15], namely *Des* and *Ptma* showed opposite gene expression patterns, and the expression region of *Ccnd2* overlaps with that of *Des* and *Ptma* (Additional file 1: Fig. S3).

### Developmental analysis of human pancreatic organoids by eSPRESSO

**Fig. 4:**
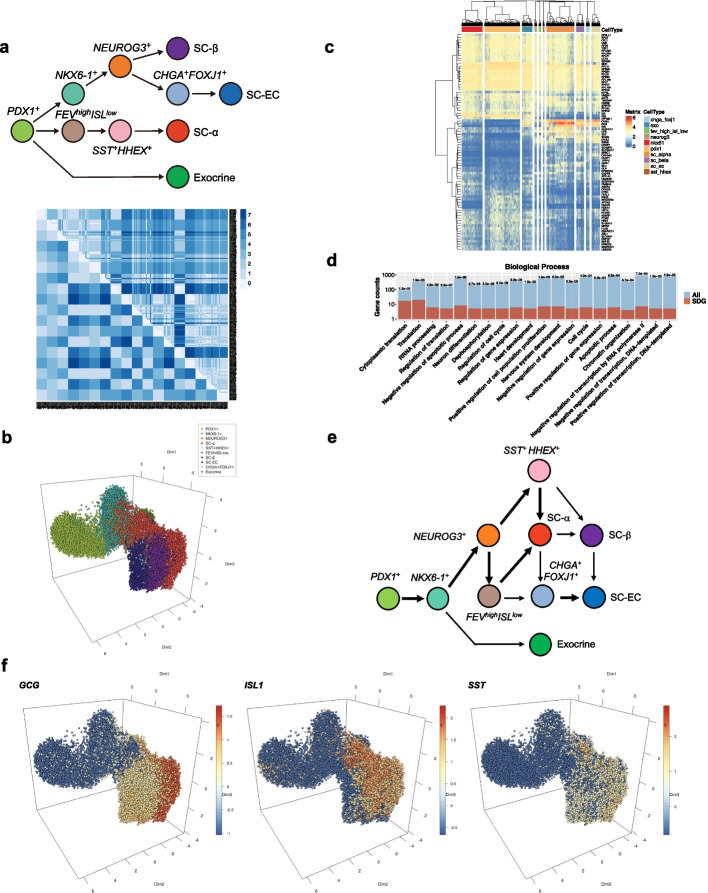
Application of eSPRESSO to detect temporal discriminator genes (TDGs) in human developmental pancreas. **a** Graph representation of cell type differentiation model and a topological distance map of cells for the original (lower left) and resultant (upper right) clusters. **b** 3D temporal reconstruction of cells with TDGs using UMAP for cell types. **c** Gene expression heatmap for the optimized TDGs. **d** GO mapping of TDGs in Biological Process. The *p*-values determined by hypergeometric distribution statistics are shown on top of bars. **e** Derived differentiation relationships of cell types from **b** by graphical lasso based on covariances of gene expressions among cell types. **f** Gene expression gradients in reconstructed temporal structure for the top three most important genes in VKO analysis

One of the powerful characteristics of eSPRESSO is demonstrated by the analysis of organs during the developmental processes. We used scRNA-seq data of human pancreatic organoids from stages 3, 4, 5, and 6 by Veres et al. [47], and attempted to reconstruct developmental or temporal structures using cells from all stages. We first combined data from all four stages to generate a coarse topological guideline of ten cell types (*PDX1*^+^, *NKX6-1*^+^, *NEUROG3*^+^, SC-α, *SST*^+^*HHEX*^+^, *FEV*^high^*ISL*^low^, SC-β, SC-EC, *CHGA*^+^*FOXJ1*^+^, and Exocrine) on the basis of the developmental model proposed in Figure 5 in the original paper [47] (Fig. 4a). The eSPRESSO clustering result showed very low topology reproducibility, namely, the average of the maximum scores of ten runs is 1.15 (accuracy: 0.76 + ARI: 0.39) (Table 1). In this case, the accuracy, which indicates topological relevance, may be sufficient, although not perfect, the ARI is unexpectedly low, which implies poor clustering results (Fig. 4b and Additional file 9: Movie S5). This is probably due to the data incongruity problem because many of the cells are assigned to the same cell type for different stages (such as SC-α and *SST*^+^*HHEX*^+^ in stages 4, 5, and 6; and SC- β, SC-EC, *SST*^+^*HHEX*^+^, *CHGA*^+^*FOXJ1*^+^, and Exocrine in stages 5 and 6) by the original paper. There are 83 consensus temporal discriminator genes (TDGs), including pancreas-specific genes such as insulin (*INS*) and insulin gene enhancer protein ISL-1 (*ISL1*) [48] (Fig. 4c, d and Additional file 10: Table S4). The VKO analysis indicated that proglucagon (*GCG*), *ISL1*, and somatostatin (*SST*) were the most influential genes for pancreas development (Additional file 11: Table S5). Indeed, *GCG* is expressed in alpha cells [49], *ISL1* is involved in embryogenesis of the islets of Langerhans [50], and SST proteins are produced in delta cells [51] (Fig. 4f and Additional file 9: Movie S5). In the reconstructed model of the human developmental pancreas using novoSpaRc, no significant differences were observed in the gene expression patterns of *GCG*, *ISL1*, and *SST*. In addition, subtle gene expression gradients, as shown by eSPRESSO were not observed using novoSpaRc (Additional file 1: Fig. S4).

Although the task was highly challenging, we devised a method for inferring a possible developmental model from the eSPRESSO results. We used consensus TDGs to generate a gene expression matrix for all the cells (83 genes by 20,205 cells). We then reduced the matrix to the original 18 cell domains or types (separated by individual stages) by taking the average within each cell domain. Using this reduced matrix, we inferred a sparse graphical Gaussian model (GGM) of cell type network based on gene expression covariance matrix using EBICglasso in qgraph package in R [52]. Considering the directions of the developmental stages, we further reduced the original 18 cell type network into a ten cell type developmental model by aggregating the same cell type from different stages if they have 0.2 or more partial correlations (Fig. 4e and Additional file 1: Fig. S5). Interestingly, the resultant developmental model was significantly different from the original model. For example, *NEUROG3*^+^ cells are precursors of SC-β and SC-EC in the coarse guideline model, but these cells are also the precursors of SC-α in the inferred model. Similarly, *NKX6-1*^+^ cells are the precursors of *NEUROG3*^+^ cells in both the coarse and inferred models, but they are also the precursors of Exocrine cells in the inferred model. These results were attributed to the actual gene expression gradients of the 83 TDGs. Indeed, a review paper on human pancreatic development indicated that *NEUROG3*^+^ cells can be the precursors of both SC-α and SC- β and that *NKX6-1*^+^ cells can be the precursors of duct (exocrine) cells [53]. Interestingly, however, scRNA-seq analysis using *Pdx-1-GFP* or other gene transgenic mouse lines to trace the pancreatic lineage revealed two α-cell generation pathways that directly generate α -cells from early *Pdx-1*^+^ cells, called the first wave, in addition to the known late α—and β-lineage differentiation [54].

## Discussion and conclusions

We developed a computational method that combines cell clustering using stochastic-SOM under topological constraints and gene set optimization using MCMC calculations. In general, the reproducibility of the known topology of cell domains is successful (Table 1), and this method may innovatively add more information to the existing clustering method by finding spatio–temporally distributed discriminator genes (SDGs or TDGs). The S(T)DGs identified by eSPRESSO showed large overlaps with the cluster biomarkers selected by Seurat [55], and all of the S(T)DGs were contained in the cluster biomarkers in some cases (Additional file 1: Fig. S6). Because the number of Seurat’s marker genes in the default settings is much higher than the number of S(T)DGs, the results make sense. As expected, most of the S(T)DGs were found to be statistically significant in Seurat with the adjusted *p*-values calculated by the Wilcoxon rank sum test between one domain and the other domains. (Additional file 1: Fig. S6). However, some genes did not show sufficient statistical significance, meaning that eSPRESSO was able to identify gene sets that were difficult to obtain by differentially expressed gene analysis with a simple statistical test. In addition, eSPRESSO was able to infer the developmental architecture of cell trajectories even with limited knowledge or coarse topological guidelines of the developmental model. Using in silico VKO analysis with eSPRESSO, it may be also useful to investigate the effects of individual genes on the topological structure, which may be difficult or impossible to accomplish in some cases in real experiments.

As mentioned in the background, direct experimental methods for the 3D reconstruction of tissues such as highly multiplexed FISH or direct spatially resolved transcriptomics have been proposed [5–7]. The primary purpose of these methods is to map or reconstruct cellular locations using gene expression data. Although these methods can contribute to detecting differentially expressed genes among distinct tissue or cell domains, they do not consider tissue topology, and thus, may miss the detection of spatially contributing genes that show expression gradients across neighboring cell domain networks. eSPRESSO may complement existing spatial transcriptomics methods by detecting gradiently or globally expressed genes that contribute to cell domain network organizations in a spatio–temporal manner. We would like to emphasize here that ARI may be high, but accuracy may be low for unstructured or complex tissues, but the results of human heart (PCW 9) analysis also show that eSPRESSO is effective in reconstructing the spatial relationship between domains even when the topology information is limited. To investigate the topological limitation of eSPRESSO, we performed computational experiments using synthetic single-cell datasets generated by the dyngen package [56]. Owing to the high calculation cost, we tested five of 14 dyngen single-cell transcription factor network models: *linear*, *cycle*, *bifurcating*, *branching*, and *disconnected* backbones [56], and generated their cellular topology graphs (Additional file 1: Figs. S7–10). eSPRESSO was able to reproduce the input topologies with near-perfect accuracy (i.e., the mean values of maximum accuracies and maximum ARIs were higher than 0.99 and 0.97, respectively) with SDGs of various expression gradient patterns regardless of the input topology graphs (Additional file 1: Figs. S9 and S10). Furthermore, when we created ten modified graphs for each dataset by randomly reversing the presence or absence of edges for each vertex pair, we observed that eSPRESSO is robust in general, that is, the mean maximum scores were higher than 1.85 for a low input cellular topology misspecification rate of 10% for all backbone configuration, and higher than 1.90 even for a high input misspecification rate of 50% except for *bifurcating* or *branching* backbone configuration (Additional file 1: Fig. S10).

eSPRESSO also has several benefits in the analysis of human organ development, where many genes give rise to homozygous lethality in knockout experiments. Although animal studies have presented evidence of candidate lethal genes in humans, it is not possible to reproduce gene knockout organs in humans because of ethical and technical reasons. Human organoids derived from the induced pluripotent stem (iPS) or embryonic stem (ES) cells show promise as an alternative approach for mimicking gene knockout organs in the human body. However, the occurrence of abnormalities is often stochastic and affected by other internal or environmental factors, and a large number of repeated experiments are sometimes required to reproduce the phenotypes [57]. In contrast, although our in silico VKO method is still in the preliminary stage, it does not require any fine-tuned protocols and reproduces the same results with a low calculation cost. In addition, such virtual experiments are possible not only for VKO experiments but also for virtual knock-in (VKI) experiments, and both are a promising alternative to costly in vitro and in vivo experiments, because the experiments can be performed regardless of the number or combination of knock-in and knock-out genes.

SOM learning, first proposed by Kohonen and colleagues [30], is an established method in the field of clustering. They introduced graph-based SOM clustering, but this method has few biological applications [29]. The original SOM learning monotonically decreases the learning regions and efficiency, which causes problems in the clustering resolution in the later phase. We found that efficient scheduling for learning is necessary for practical application to current single-cell transcriptomics datasets. We introduced stochastic-SOM, which has similar behavior to the Gibbs sampling approach where SOM clustering is continued by random sampling even in the late learning phase. This improvement dramatically enhanced the clustering resolution and enabled efficient implementation of graph-based SOM clustering.

Finally, we would like to stress that our current algorithms and analysis pipeline reported herein are still primitive and limited, and many improvements are necessary. First, there are cases in which certain difficulties remain in the creation of topology graphs. For a one-dimensional layered structure, such as a mouse brain (ALM and VISp), or a relatively simple tissue structure, such as a mouse embryo, it is possible to easily create a topology graph based on general biological knowledge. However, it is sometimes difficult to accurately represent complex topological structures, such as the heart, in graphs. In fact, for the human heart dataset, we created a dense model that considers all indefinite adjacency relations as connected and a sparse model that considers those as not connected. In addition, graph resolution was not determined by itself, but depended on the purpose of the user’s analysis. As an example guideline for automatic determination, it may be possible to use the clustering results from Louvain’s method [58].

Second, the extraction of initial gene candidates is solely dependent on the Random Forest algorithm that detects feature genes by the Boruta package in R and may miss important SDGs or TDGs that overlap categories or cell domains in distant branches or leaves. Although the input genes to Boruta were selected on the basis of the frequency of nonzero expression in all cells and the standard deviation in the computational experiments, we observed no significant change in the structure reconstruction performance in our datasets even when highly variable genes selected simply by Seurat were used as the input genes to Boruta (Additional file 1: Fig. S11 and Additional file 12: Table S6). The replica exchange MCMC calculation is also a suboptimal approach, although it enhances combinatorial optimization to allow quick convergence to the global optimum, which sometimes fails to find the best gene set to reproduce the topology using SOM clustering. Furthermore, the selected feature gene sets differ across runs. Therefore, in the computational experiments, genes detected as SDGs three or more times in ten runs were used as consensus SDGs in subsequent analyses. This experimental approach has contributed to improving the reliability of SDGs even for single-cell and mini-bulk data, which are often noisy.

Third, it is difficult for eSPRESSO to determine whether the differences in gene expression patterns in SDGs are due to spatial distributions or cell type differences. To address this limitation, a more effective algorithm that detects hierarchical relationships of gene expression profiles should be developed. Furthermore, the computation time in this study ranged from 1 to 10 days on 2.60 GHz E5-2670 Intel Xeon CPU, depending on the amount and complexity of data. We expect that more efficient and fine-grained algorithms for finding the global optimum in an acceptably short time may replace the modules in our pipeline in the future.

## Methods

### Data collection and preprocessing

To confirm the performance of eSPRESSO, 14 datasets of human and mouse transcriptomes were collected from seven papers [10, 36, 39–41, 47, 59]. Each dataset was used as a log10-transformed expression profile, and genes whose expression values are greater than 1.0 in at least two samples and whose standard deviations across all samples are greater than 0.05 were extracted. Adjacent matrices of spatial domains were constructed on the basis of original papers and biological knowledge by the authors of this paper.

### Mm embryo (E7.0)

Peng et al. collected transcriptome profiles of embryo sections by laser microdissection [59]. We downloaded and used E1 dataset from GEO (GSE65924) [60], which is one of the triplicates of single embryos. The dataset contains 41 sections (~ 20 cells per sample) with four spatial domains (d1: anterior, d2: lateral-distal, d3: lateral-proximal, and d4: posterior). The expression values were saved as fragments per kilobase of exon per million mapped reads (FPKM).

### Mm embryo (E7.5)

Peng et al. collected transcriptome profiles of embryo sections at various developmental stages by laser microdissection [36]. We downloaded reference samples of E7.5 from GEO (GSE120963). The dataset contains 83 sections (20–40 cells per sample) with nine spatial domains (Ect1–3: ectoderm, PS: primitive streak, MA: anterior mesoderm, MP: posterior mesoderm, E1–3: endoderm). To simplify the domain structure, we integrated E1–3 into En as a single domain. The expression values were saved as FPKM.

### Mm brain (ALM and VISp)

Tasic et al. collected transcriptome profiles of two regions of adult mouse cortex: anterior lateral motor cortex (ALM) and primary visual cortex (VISp) [39]. We downloaded exon count datasets of ALM and VISp from GEO (GSE115746), respectively. For both datasets, we extracted 3809 and 7049 single cells belonging to clusters with distinct layer numbers (L2/3, L4, L5, L6, and L6b), respectively, where the L6b cells were merged to the L6 cluster. ALM consists of three spatial domains of L2/3, L5, and L6, whereas VISp consists of four spatial domains of L2/3 to L6. The expression values were saved as raw count data and transformed into counts per million mapped reads (CPM) values when analyzed.

### Mm heart (E7.75, E8.25, and E9.25)

de Soysa et al. collected transcriptome profiles of mouse heart at three developmental stages: E7.75, E8.25, and E9.25 [41]. We downloaded source data of all developmental stages from the supplementary files of the original paper. In addition, we requested the expression profiles of sinus venosus (SV) and atria at E9.25 to the authors of the original paper for integration with the downloadable E9.25 data. For E7.75 data, the anterior heart field (AHF), the left ventricle (LV), atria (Atrial), and the posterior second heart field (pSHF) of wild type were extracted and the total number of single cells was 1259. For E8.25 and E9.25 data, 3331 and 3911 single cells of AHF, the SHF-derived outflow tract (OFT), the right ventricle (RV), LV, atria (Atrial), SV, and pSHF were extracted, respectively. The expression values were saved as log-transformed unique molecular identifier (UMI) counts.

### Mm liver

Halpern et al. collected transcriptome profiles of mouse liver and estimated their lobule coordinates by a panel of zonated landmark genes [10]. The authors provided a single-cell gene expression profile and a posterior probability matrix showing the probabilities of being the original layer for each single cell, and they can be downloaded as supplementary files of the original paper. In order to simplify the input data, we determined the layer that gives the maximum probability as the original layer for each single cell. This dataset consists of 1415 single cells with nine domains (layers 1–9). The expression values were saved as raw UMI counts and transformed into CPM when analyzed.

### Hs pancreas organoid (S3–S6)

Veres et al. collected transcriptome profiles of human pancreas at four differentiation stages [47]. We downloaded the preprocessed gene expression profile of Protocol x1 from four stages 3–6 (S3–S6) from GEO (GSE114412), where profiles of replications were removed. Dataset S3 consists of 5955 single cells of PDX1^+^ progenitors (pdx1). For dataset S4, the expression profiles consist of 5273 single cells with five domains: NKX6-1^+^ progenitors (nkx61), NEUROG3^+^ progenitors (neurog3), SC-$\alpha$ (sc_alpha), SST^+^HHEX^+^ (sst_hhex), and FEV^high^ISL^low^ (fev_high_isl_low). Datasets S5 and S6 consist of 3926 and 5051 single cells from six domains: SC-$\beta$ (sc_beta), SC-$\alpha$ (sc-alpha), SC-EC (sc-ec), CHGA^+^FOXJ1^+^ (chga_foxj1), SST^+^HHEX^+^, and Non-endocrine (exo). These datasets were merged into one dataset of 20,205 single cells with complementing missing gene expression values from raw count data provided by GEO (GSE114412). The expression values of these datasets were saved as UMI counts and transformed into CPM when analyzed.

### Hs heart (PCW 4.5–5, PCW 6.5, and PCW 9)

Asp et al. collected transcriptome profiles of human heart at three developmental stages in the first trimester: post-conception weeks (PCWs) 4.5–5, 6.5, and 9 [40]. Each expression profile and the corresponding metadata are downloadable from a data repository (https://www.spatialresearch.org). The spatial transcriptome data of PCW 4.5–5, PCW 6.5, and PCW 9 consist of 238, 1515, and 1358 spots of tissue sections. The PCW 4.5–5 and PCW 6.5 data cover eight domains: compact ventricular myocardium (CVM), trabecular ventricular myocardium (TVM), atrial myocardium (AM), outflow tract and large vessels (OTLV), atrioventricular mesenchyme and valves (AMV), mediastinal mesenchyme and vessels (MMV), cavities with blood and immune cells (CBIC), and epicardium (Epi), whereas the PCW 9 data cover seven domains (the same domains as above except CBIC).

### Overview of eSPRESSO

Our previously reported SPRESSO [26], which is a 3D reconstruction method using SOM clustering and GO-based feature gene selection, has achieved a high success rate in the 3D reconstruction of mouse gastrula structure and shown a remarkable ability to identify SDGs that contribute to differentiation and tissue morphogenesis. However, it projects the mouse gastrula structure into a simple cubic structure composed of four domains and is therefore inapplicable to more complex tissues. In addition, whereas the feature gene selection using GO has enabled us to search feature genes on the basis of their functions, it is difficult to expand the search space to combinations of GOs from the perspective of computation time because more than 40,000 GOs have been defined.

eSPRESSO is able to overcome the limitations of SPRESSO through the introduction of graph-based SOM clustering and gene set optimization by the MCMC framework. The graph-based SOM clustering enables the reconstruction of any topology of cell domains in tissues, as long as they can be drawn as network diagrams or graphs, thereby greatly expanding the applicability of eSPRESSO. Meanwhile, the gene set optimization by the MCMC framework enables more flexible search for feature gene sets that is not restricted to the definitions of GOs while limiting the search space to promising areas by combining with Boruta, a feature gene selection method. Details of graph-based SOM clustering and gene set optimization by the MCMC framework are described in the following sections.

### Stochastic self-organizing map (stochastic-SOM) clustering

The self-organizing map (SOM) is an unsupervised clustering method proposed by Kohonen [30]. In general, SOM projects input high-dimensional data onto a limited number of output classes or units, so that different units with similar centroid vectors are placed close to each other in a mapping layer that is usually given in a 2D plane. Let ${\varvec{X}} = \left( {{\varvec{x}}_{1} ,{\varvec{x}}_{2} , \ldots ,{\varvec{x}}_{n} } \right)$ be a set of input samples with the $p$-dimensional vectors, i.e., ${\varvec{x}}_{j} = \left( {x_{j1} ,x_{j2} , \ldots ,x_{jp} } \right)$ $\left( {j = 1, 2, \ldots , n} \right)$. The mapping layer consists of $k$ units, and their centroid vectors ${\varvec{m}}_{i} = \left( {m_{i1} , m_{i2} , \ldots ,m_{ip} } \right)$ $\left( {i = 1, 2, \ldots , k} \right)$ are randomly initialized and assigned to each unit. The similarity between input sample $j$ and all units $i$ is defined by the Euclidean distance. First, the SOM algorithm finds the unit $c$ with the highest similarity according to the following equation as the best matching unit (BMU).

$$c = {\text{arg}}\mathop {\min }\limits_{{i \in \left\{ {1, \ldots ,k} \right\}}} \left\{ {\left\| {{\varvec{x}}_{j} - {\varvec{m}}_{i} \left( t \right)} \right\|} \right\},$$

where $\left\| \cdot \right\|$ denotes the Euclidean distance, or norm of a vector, and $t$ is time step. The centroid vector ${\varvec{m}}_{i} \left( t \right)$ of all units of the mapping layer at time $t$ is updated by the following equations.

$${\varvec{m}}_{i} \left( {t + 1} \right) = {\varvec{m}}_{i} \left( t \right) + h_{ci} \left( t \right)\left( {{\varvec{x}}_{j} - {\varvec{m}}_{i} \left( t \right)} \right),$$

$$h_{ci} \left( t \right) = \alpha \left( t \right){\text{exp}}\left( { - \frac{{\left\| {{\varvec{r}}_{c} - {\varvec{r}}_{i}^{2} } \right\|}}{{2\sigma^{2} \left( t \right)}}} \right),$$

where $h_{ci} \left( t \right)$ is a neighborhood function that determines how much ${\varvec{m}}_{i} \left( t \right)$ receives the learning influence of ${\varvec{x}}_{j}$ when it is updated. $\alpha \left( t \right)$ and $\sigma \left( t \right)$ are the learning rate parameter and a function defining the radius of the neighboring region, respectively. In addition, ${\varvec{r}}_{c}$ and ${\varvec{r}}_{i}$ are the position vectors in the mapping layer of units $c$ and $i$. The SOM algorithm repeats updates of ${\varvec{m}}_{i}$ until the learning step $t$ reaches $T,$ which is given as a parameter for all input samples $j$.

In the general SOM clustering, its result is affected by the order in which the samples are input. To eliminate this effect, the batch-learning SOM was also proposed [30]. In the batch-learning SOM, ${\varvec{m}}_{i} \left( t \right)$ is updated only after all samples are given by the following equations.

$$c_{j} \left( t \right) = {\text{arg}}\mathop {\min }\limits_{{i \in \left\{ {1, \ldots ,k} \right\}}} \left\{ {\left\| {{\varvec{x}}_{j} - {\varvec{m}}_{i} \left( t \right)} \right\|} \right\},$$

$${\varvec{m}}_{i} \left( {t + 1} \right) = \frac{{\mathop \sum \nolimits_{j = 1}^{n} h_{{c_{j} \left( t \right)i}} \left( t \right){\varvec{x}}_{j} }}{{\mathop \sum \nolimits_{j = 1}^{n} h_{{c_{j} \left( t \right)i}} \left( t \right)}}.$$

The general SOM learning often converges to local minima in early steps if the number of units in the mapping layer is extremely small. In order to increase the possibility of escaping from the local minima and reaching the global maxima, a stochastic-SOM that introduces a random variable into the neighborhood function has been proposed, which makes the learning converge gradually [26]. The neighborhood function of the stochastic-SOM is

$$h_{ci} \left( t \right) = {\text{exp}}\left( { - \frac{{{\text{rnd}}\left[ {0.5, 1} \right) \cdot \left\| {{\varvec{r}}_{c} - {\varvec{r}}_{i}^{2} } \right\|}}{{2\sigma^{2} \left( t \right)}}} \right),$$

where ${\text{rnd}}\left[ {0.5, 1} \right)$ is a function that generates uniform random values of at least 0.5 and less than 1.0.

### Graph-based stochastic-SOM clustering

A graph $G = \left( {V,E} \right)$ is a pair of finite non-empty set $V$ and finite set $E \subseteq V \times V$. The elements $u,v \in V$ of graph $G$ are called vertices, and the elements $e = \left\{ {u,v} \right\} \in E$ are called edges. The sets of vertices and edges of graph $G$ are denoted as $V\left( G \right)$ and $E\left( G \right)$, and their numbers are denoted as $\left| {V\left( G \right)} \right|$ and $\left| {E\left( G \right)} \right|$, respectively. A path on $G$ is a non-empty graph $P\left( G \right) = \left( {V,E} \right)$, where $V = \left\{ {v_{i} ,v_{i + 1} , \ldots ,v_{j} } \right\}$ and $E = \left\{ {\left\{ {v_{i} ,v_{i + 1} } \right\},\left\{ {v_{i + 1} ,v_{i + 2} } \right\}, \ldots ,\left\{ {v_{j - 1} ,v_{j} } \right\}} \right\}$, and all $v_{k}$ values are distinct. The distance $d_{G} \left( {u,v} \right)$ between two vertices $u$ and $v$ on $G$ is given by the length (i.e., the number of edges) of the shortest path between $u$ and $v$. Graph $G$ is often represented by square matrix $A\left( G \right) = \left[ {a_{ij} } \right] \left( {i,j = 1,2, \ldots ,\left| {V\left( G \right)} \right|} \right)$ that shows the adjacency between vertices; this matrix is called an adjacency matrix, where $a_{ij} = 1$ if $\left\{ {v_{i} ,v_{j} } \right\} \in E\left( G \right),$ otherwise $a_{ij} = 0$ for the vertices $v_{i}$ and $v_{j}$ corresponding to $i$ and $j$, respectively. Note that $a_{ij} = 0$ when $i$ and $j$ are identical because self-loops are not assumed.

To improve the performance of stochastic-SOM, we newly propose graph-based SOM (graph-SOM) clustering. In the graph-SOM, the mapping layer is given by graph $G$ represented by the adjacency matrix $A\left( G \right)$. Although in the general SOM, the distance between units $i$ and $j$ in the mapping layer is computed by the Euclidean distance $\left\| {{\varvec{r}}_{i} - {\varvec{r}}_{j} } \right\|$ between the corresponding position vectors ${\varvec{r}}_{i}$ and ${\varvec{r}}_{j}$, each unit corresponds to a vertex on $G$ and the distance between the units is given by distance $d_{G} \left( {v_{i} ,v_{j} } \right)$ between the vertices $v_{i}$ and $v_{j}$ on $G$ in the graph-SOM. Therefore, the neighborhood function $h_{ci} \left( t \right)$ at time $t$ of the stochastic graph-SOM is given by the following equation.

$$h_{ci} \left( t \right) = {\text{exp}}\left( { - \frac{{{\text{rnd}}\left[ {0.5, 1} \right) \cdot d_{G} \left( {v_{c} ,v_{i} } \right)^{2} }}{{2\sigma^{2} \left( t \right)}}} \right).$$

### Evaluation of graph-SOM clustering results

The clustering result of the stochastic graph-SOM is evaluated on the basis of two criteria: prediction accuracy ($Accuracy$) and adjusted Rand index (ARI). The score function is defined by the following equation:

$$Score = Accuracy + a \cdot ARI,$$

where $a$ is a constant parameter that adjusts the weight of *ARI* for *Accuracy* and $a = 1.0$ is employed as the default setting of eSPRESSO.

### Prediction accuracy: *accuracy*

For a pair of cell samples $c_{i}$ and $c_{j}$, let $d_{i}$ and $d_{j}$ be the true domains to which they belong, and let $\hat{d}_{i}$ and $\hat{d}_{j}$ be the domains to which they are estimated to belong by the stochastic graph-SOM. Here, assuming that $s_{xy}$ is an element of the adjacency matrix $A\left( G \right)$ corresponding to the input graph $G$, the prediction score $s_{ij}$ for a pair $\left\{ {c_{i} ,c_{j} } \right\}$ is given by the following equation

$$s_{ij} = \left\{ {\begin{array}{*{20}c} {1 \left( {a_{{d_{i} d_{j} }} = a_{{\hat{d}_{i} \hat{d}_{j} }} } \right)} \\ {0 \left( {a_{{d_{i} d_{j} }} \ne a_{{\hat{d}_{i} \hat{d}_{j} }} } \right)} \\ \end{array} } \right..$$

Therefore, the prediction accuracy $Accuracy$ for all cell sample pairs is defined by the following equation

$$Accuracy = \frac{{\mathop \sum \nolimits_{i,j}^{{\left( {\begin{array}{*{20}c} n \\ 2 \\ \end{array} } \right)}} s_{ij} }}{{\left( {\begin{array}{*{20}c} n \\ 2 \\ \end{array} } \right)}},$$

where *n* is the total number of cell samples.

### Adjusted Rand index (ARI)

The adjusted Rand index (ARI) measures the similarity between two clustering results [61]. Let ${\mathcal{X}} = \left\{ {{\mathcal{X}}_{1} ,{\mathcal{X}}_{2} , \ldots ,{\mathcal{X}}_{m} } \right\}$ and ${\mathcal{Y}} = \left\{ {{\mathcal{Y}}_{1} ,{\mathcal{Y}}_{2} , \ldots ,{\mathcal{Y}}_{m} } \right\}$ be families of sets of cell samples, where $m$ is the number of domains. The overlap of cell samples between ${\mathcal{X}}_{i}$ and ${\mathcal{Y}}_{j}$ is denoted by $n_{ij} \left( { = \left| {{\mathcal{X}}_{i} \cap {\mathcal{Y}}_{j} } \right|} \right)$. The number of cell samples belonging to ${\mathcal{X}}_{i}$ (resp. ${\mathcal{Y}}_{j}$) can be represented by using $n_{ij}$ as $a_{i} = \mathop \sum \limits_{j = 1}^{m} n_{ij}$ (resp. $b_{j} = \mathop \sum \limits_{i = 1}^{m} n_{ij}$). Therefore, ARI can be defined by the following equation:

$$ARI\left( {{\mathcal{X}},{\mathcal{Y}}} \right) = \frac{{\mathop \sum \nolimits_{i,j} \left( {\begin{array}{*{20}c} {n_{ij} } \\ 2 \\ \end{array} } \right) - \left[ {\mathop \sum \nolimits_{i} \left( {\begin{array}{*{20}c} {a_{i} } \\ 2 \\ \end{array} } \right)\mathop \sum \nolimits_{j} \left( {\begin{array}{*{20}c} {b_{j} } \\ 2 \\ \end{array} } \right)} \right]/\left( {\begin{array}{*{20}c} n \\ 2 \\ \end{array} } \right)}}{{\frac{1}{2}\left[ {\mathop \sum \nolimits_{i} \left( {\begin{array}{*{20}c} {a_{i} } \\ 2 \\ \end{array} } \right) + \mathop \sum \nolimits_{j} \left( {\begin{array}{*{20}c} {b_{j} } \\ 2 \\ \end{array} } \right)} \right] - \left[ {\mathop \sum \nolimits_{i} \left( {\begin{array}{*{20}c} {a_{i} } \\ 2 \\ \end{array} } \right)\mathop \sum \nolimits_{j} \left( {\begin{array}{*{20}c} {b_{j} } \\ 2 \\ \end{array} } \right)} \right]/\left( {\begin{array}{*{20}c} n \\ 2 \\ \end{array} } \right)}},$$

where $n$ is the total number of cell samples. Here, assuming that ${\mathcal{X}}$ and ${\mathcal{Y}}$ are the true family of domains and the family of domains estimated by the stochastic graph-SOM, respectively, the similarity between the true domain classification and the estimated domain classification can be computed by ARI.

### Optimization of cluster allocation of graph-SOM

When applying eSPRESSO to tissues whose topological structure is unstructured or complex, Graph-SOM results often have high ARI and low prediction accuracy because ARI itself calculates the clustering accuracy without considering the cell-to-cell adjacency between the clusters. Therefore, eSPRESSO introduces an operation to optimize the allocation of clusters to the vertices on graph-SOM by swapping the allocation while preserving the members of each cluster. The swapping operation occurs on the basis of the simulated annealing (SA) strategy [62]. A pair of clusters $i$ and $j$ is selected to be swapped according to the selection probability $p_{i,j}$, which is weighted by the post-swapping accuracy computed in advance.

$$p_{i,j} = \frac{{\left( {\exp \left( {z_{i,j} } \right)} \right)^{c} }}{{\mathop \sum \nolimits_{k = 1}^{m - 1} \mathop \sum \nolimits_{l = k + 1}^{m} \left( {\exp \left( {z_{k,l} } \right)} \right)^{c} }},$$

where $z_{i,j} = \frac{{acc_{i,j} - \mu }}{\sigma }$, and $\mu$ and $\sigma$ are the mean value and the standard deviation of the accuracy $acc_{i,j}$ for the case that clusters $i$ and $j$ are swapped. $m$ is the number of clusters and $c$ is a constant parameter defined by $c = \sqrt {m/2}$. Once a pair of swap candidates is selected, whether or not the swap is adopted is determined according to the following adoption probability $p_{SA}$ based on the SA strategy.

$$p_{SA} = \left\{ {\begin{array}{*{20}c} {1 \left( {\Delta f \le 0} \right)} \\ {exp\left( {\frac{ - \Delta f}{{T_{t} }}} \right) (\Delta f > 0)} \\ \end{array} } \right.,$$

where $\Delta f$ is the difference in accuracy before and after the cluster swapping (i.e., $a_{before} - a_{after}$). $T_{t}$ is a temperature parameter at the $t$-th learning step, which exponentially decreases from 1.0 to 0.001. Finally, when clusters $i$ and $j$ are swapped, the centroid vectors of the corresponding vertices on the graph-SOM are also swapped.

### Optimization of gene set by Markov chain Monte Carlo (MCMC) framework

In order to obtain the spatial discriminator genes (SDGs), eSPRESSO employs Random Forest-based feature gene selection method Boruta [63] and replica exchange Markov chain Monte Carlo-based gene set optimization.

### Feature gene selection by Boruta

Kursa and Rudnicki proposed the Boruta method for a Random Forest-based algorithm for feature selection [63]. One of the properties of Boruta is to classify features into three classes: *confirmed*, *tentative*, and *rejected*, rather than order them. In eSPRESSO clustering, we selected from 64 to 2377 *confirmed* and *tentative* genes as features for each dataset by increasing the value of parameter `maxRuns` in the Boruta package of programming language R.

### Replica exchange MCMC optimization

After obtaining the feature genes by Boruta, eSPRESSO searches for the optimum combination of the feature genes by the replica exchange MCMC framework and then outputs the SDGs. The replica exchange MCMC is an extended algorithm of MCMC for improving sampling efficiency [37]. In a general simulated annealing (SA) algorithm [62], which is one of the optimization algorithms based on MCMC sampling, there is only one temperature parameter that determines whether to adopt or reject the newly obtained sample, and the probability of being adopted is relatively high even for samples with a large energy difference when the temperature is high. However, it is difficult to get out of the local minima at a low temperature. As a result, the probability of being rejected increases and sampling efficiency decreases. In the replica exchange MCMC, multiple systems called replicas with different parameters are simulated at the same time, and the states of the replicas are exchanged between different temperatures according to the following exchange probability:

$$p = \mathop {\min }\limits_{ } \left( {1,{\text{exp}}\left( {\left( {E_{i} - E_{j} } \right)\left( {\frac{1}{{T_{i} }} - \frac{1}{{T_{j} }}} \right)} \right)} \right),$$

where $E_{k}$ and $T_{k}$ are the energy and the temperature of replica $k$, respectively. By updating the variables of each replica at their respective temperatures and moving on the temperature axis at the same time, its long-term behavior can be regarded as a random walk.

In eSPRESSO, sampling in each replica is done by the following MCMC sampling algorithm.

(Step 1): Let $G$ be an empty set and initialize the score of $G$ with negative infinity and let $G_{b}$ be the feature gene set obtained by Boruta.
(Step 2): Select $n^{\prime}$ genes from $G_{b}$ at uniformly random (i.e., according to equal probability) and let $G\prime$ be the set of $n^{\prime}$ genes.
(Step 3): Repeat the following procedures $T$ times.
  i. Generate candidate gene sets $\mathcal{G}= \left\{ {G_{cand}^{1} ,G_{cand}^{2} , \ldots ,G_{cand}^{N} } \right\}$ by $G_{cand}^{i} = G^{\prime} \cup \left\{ {g_{i} } \right\}$ and $G_{cand}^{j} = G\prime \setminus \left\{ {{\text{g}}_{{\text{j}}} } \right\}$ for all $g_{i} \in G_{b} \setminus G^{\prime }$ and all $g_{j} \in G^{\prime}$.
  ii. Remove gene sets $G_{cand}^{i}$, which are already sampled.
  iii. If $\mathcal{G}$ is an empty set, replace gene $g_{j} \in G^{\prime}$ with another gene $g_{k} \in G_{b} \setminus \left\{ {g_{j} } \right\}$ and add them to $\mathcal{G}$.
  iv. For all $G_{cand}^{i}$, execute the stochastic graph-SOM clustering and compute the scores of the clustering results (see the Section Evaluation of graph-SOM clustering results for more details).
  v. Define selection probability $p_{i}$ for $G_{cand}^{i}$ by
    $$p_{i} = \frac{{\left( {{\text{exp}}\left( {z_{i} } \right)} \right)^{c} }}{{\mathop \sum \nolimits_{i = 1}^{N} \left( {{\text{exp}}\left( {z_{i} } \right)} \right)^{c} }},$$
    where $z_{i} = \frac{{s_{i} - \mu }}{\sigma }$, and $\mu$ and $\sigma$ are the mean value and the standard deviation of the scores $s_{i}$ of $G_{cand}^{i}$, respectively. $c$ is a constant parameter and $c = \sqrt {\left| \mathcal{G} \right|/2}$ is employed in the computational experiments in this paper.
  vi. Determine whether to adopt or reject $G_{cand}^{x}$ according to the adoption probability $p_{SA}$ of the SA strategy.
    $$p_{SA} = \left\{ {\begin{array}{*{20}c} {1 \left( {\Delta f \le 0} \right)} \\ {exp\left( {\frac{ - \Delta f}{{T_{t} }}} \right) (\Delta f > 0)} \\ \end{array} } \right.$$
    Note that eSPRESSO handles the maximization problem for the score $s$, so that the difference $\Delta f$ is defined by $\Delta f = - \left( {s_{x} - s} \right)$, where $s_{x}$ and $s$ are the scores of $G_{cand}^{x}$ and $G$, respectively.
  vii. Update $G^{\prime}$ by $G_{cand}^{x}$ if adopted, and update also $G$ by $G_{cand}^{x}$ if the score of $G_{cand}^{x}$ is larger than that of $G$.
(Step 4): Output $G$ as the optimized gene set.

### Gene Ontology (GO) analysis

eSPRESSO generates (sub-)optimal gene sets for topological clustering. We repeated eSPRESSO clustering with different random seeds and determined consensus spatial or temporal discriminator genes that are found three or more times in ten runs. Then, we used biomaRt package [64] in R-4.0.5 to count the numbers of consensus genes and all genes assigned to each GO term in “Biological process” and “Molecular function” categories. Using phyper function in R, we calculated *p*-values on the basis of the hypergeometric distribution model.

### Virtual knockout analysis

To analyze the dependencies of the spatial or temporal discriminator genes, we performed eSPRESSO clustering 100 times with different seeds for the same data by removing genes in a combinatorial manner, and calculated the mean score. We ranked the genes according to the decrease of the mean score to find the most to least influential genes for clustering.

### Glasso for sparse developmental network estimation

Once we obtained a gene by cell domain matrix by averaging the gene expression of consensus gene sets, we inferred a sparse graphical Gaussian model (GGM) of cell domain network based on gene expression covariance matrix using EBICglasso with gamma = 0.5 and nlambda = 1000 in qgraph package [65] in R.

### Reconstruction of spatial arrangement by novoSpaRc

To compare the gene expression gradients in models reconstructed by eSPRESSO with those obtained by another approach, we reconstructed the mouse developmental heart and human developmental pancreas by novoSpaRc [15]. We employed a circle shape as a target space with the same number of locations as the number of cell samples and reconstructed the tissues using `reconstruct` function with alpha_linear = 0 and epsilon = 5e−3, where the human developmental pancreas data were downsampled to 5000 cells in advance.

### Cluster biomarker detection by Seurat

eSPRESSO identifies SDGs or TDGs by graph-based SOM clustering and MCMC optimization. As an alternative approach, differential gene expression analysis acquires domain-specifically expressed genes without considering the adjacency between domains. To compare S(T)DGs and such domain-specifically expressed genes, we performed cluster biomarker detection by Seurat [55] using the `FindMarkers` function for all datasets. For each dataset, biomarkers with adjusted *p*-values less than 0.05 and log2-fold change greater than 0.25 were collected from each domain. Venn diagrams comparing the consensus S(T)DGs identified by eSPRESSO and the cluster biomarkers identified by Seurat were generated by the `VennDiagram` package in R. Furthermore, to clarify the statistical significance of those S(T)DGs when comparing between domains, we performed enrichment analysis of S(T)DGs for the gene lists ranked according to the adjusted *p*-values obtained by the Wilcoxon rank sum test between one domain and the other domains for each dataset by using the `fgseaMultilevel` and `plotEnrichment` functions in `fgsea` package in R.

### Synthetic single-cell datasets and topology graphs

In order to evaluate the performance of eSPRESSO, we performed additional computational experiments using synthetic single-cell datasets. Although eSPRESSO requires a set of a gene expression data and a topology graph of cell domains, as far as we know, there is no method to artificially create a topology graph on the basis of real single-cell dataset. Therefore, first, we created synthetic single-cell datasets by using `dyngen` in R package [56] with a standard procedure (i.e., applying `initialise_model` and `generate_dataset` functions), which can simulate biological data for 14 predefined transcription factor network models including *linear*, *cycle*, *bifurcating*, *branching*, and *disconnected* backbones configurations, and then generated the topology graphs of cells based on the Pearson correlation coefficients of these gene expression data. When generating the topology graphs, we first identified cell clusters by Louvain’s clustering [58] for each synthetic single-cell dataset after standard preprocessing by Seurat (i.e., applying `NormalizeData`, `FindVariableFeatures`, `ScaleData`, `RunPCA`, `FindNeighbors`, and `FindClusters` functions). Finally, we generated the topology graphs of cells using the Pearson correlation coefficients of the centroids of identified clusters, where the clustering cutoff was set between 0.70 and 0.85 so that the resulting topology graphs hold consistency, i.e., disconnected for *disconnected* dataset and connected for the other datasets.

### Impact of misspecification of input topology

In order to clarify the impact of input topology misspecification on the prediction accuracy and the ARI, we performed randomization using the synthetic topology graphs. We selected five datasets derived from typical dyngen models with *linear*, *cycle*, *bifurcating, branching,* and *disconnected* backbones from the 14 synthetic datasets created in the previous section. Then, we created ten modified disconnected graphs for *disconnected* and ten connected graphs for the other datasets by reversing the presence or absence of edges for each vertex pair according to the randomization probability $p$, which takes a value of 0.1, 0.2, or 0.5. When applying eSPRESSO, we calculated the prediction accuracy and the ARI for each synthetic dataset by using the modified topology instead of the original topology together with the synthetic expression count matrix as input.

## Supplementary Information

**Additional file 1 Fig. S1.** GO mapping of SDGs in Biological Process for mouse E7.0 gastrula embryo. The *p*-values determined on the basis of hypergeometric distribution statistics are shown on top of bars. **Fig. S2.** Topology graphs for 12 datasets used as input for eSPRESSO clustering in this study. **Fig. S3.** Gene expression gradients in reconstructed models by eSPRESSO (upper) and novoSpaRc (lower) for the top three important genes in VKO analysis of mouse developmental heart. **Fig. S4.** Gene expression gradients in reconstructed models by eSPRESSO (upper) and novoSpaRc (lower) for the top three important genes in VKO analysis of the human developmental pancreas. **Fig. S5.** GGM raw result by glasso for original 18 cell types at individual stages (left) and induced developmental model by reduced 10 cell types (right). **Fig. S6.** Venn diagrams representing consensus S(T)DGs identified by eSPRESSO and cluster biomarkers identified by Seurat, and enrichment plots of the S(T)DGs for the gene lists ranked according to the adjusted *p*-values obtained by the Wilcoxon rank sum test between one domain and the other domains. P.adj and NES denote adjusted *p*-value and normalized enrichment score, respectively. **Fig. S7** Synthetic single-cell dataset created from a dyngen model with *linear* backbone of transcription factor module configuration, and a topology graph generated from the dataset. **a** Module network of *linear* backbone generated by dyngen. **b** Transcription factor and target gene regulatory network generated by dyngen. **c** UMAP of 1,000 cells of the synthetic dataset. Numbers in the legend are the cluster numbers identified by the Louvain method. **d** Topology graph generated from the synthetic dataset. Vertex labels indicate domain names corresponding to the cluster numbers in (**c**). **Fig. S8**. Topology graphs generated from synthetic single-cell datasets of predefined 14 backbones by dyngen. The dyngen parameters for generating each dataset are indicated by Seed, random seed; #TF, the number of transcription factors; #Targets, the number of target genes; and #HK, the number of housekeeping genes. Thresholds indicate the cut-off values for generating topology graphs from the correlation networks of identified domains. **Fig. S9** Expression gradients of SDGs identified by eSPRESSO on topology graphs generated from the synthetic datasets. Vertex colors indicate gene expression levels (Z-score) of the cluster centroids. **Fig. S10.** Impact of misspecification of input topology. Each plot shows the mean values of maximum scores (= accuracy + ARI), maximum accuracies, and maximum ARIs, respectively, and 95% confidence intervals for ten experiments with increasing randomization probability. **Fig. S11.** Venn diagrams representing input genes to Boruta selected on the basis of the frequency of nonzero expression in all cells as well as the standard deviation (eSPRESSO) and highly variable genes by Seurat (Seurat).

**Additional file 2 Movie S1.** The final topological clustering result for the 83 sections using 36 consensus SDGs for mouse E7.5 gastrula embryo.

**Additional file 3 Table S1.** 66 consensus SDGs for E9.5 mouse liver and their GO enrichment analysis.

**Additional file 4 Movie S2.** The final topological clustering result for 1,415 cells with 66 consensus SDGs for mouse liver lobule structure data (left), and 1,358 spots with 70 consensus SDGs for PCW 9 human embryonic heart data (right).

**Additional file 5 Table S2.** 70 consensus SDGs for PCW 9 human heart and their GO enrichment analysis.

**Additional file 6 Movie S3.** The final topological clustering result for the 3,331 single-cells using 20 consensus SDGs for mouse E9.25 embryonic heart (top left). Topological clustering results for individual VKO experiments (*Des*^−^, *Ptma*^−^, or *Ccnd2*^−^) and multiple VKO experiments (*Des*^−^*Ptma*^−^, and *Des*^−^*Ptma*^−^*Ccnd2*^−^) are also shown.

**Additional file 7 Table S3.** 20 consensus SDGs for E9.25 mouse heart and their GO enrichment analysis.

**Additional file 8 Movie S4.** The gene expression distribution of *Des*, *Ptma* and *Ccnd2* genes. *Des* and *Ptma* are mainly up-regulated in OFT and pSHF domains, respectively, and down-regulated vice versa, whereas *Ccnd2* is mainly up-regulated in LV and pSHF domains but down-regulated in the middle of both, i.e., in Atrial domain.

**Additional file 9 Movie S5.** The final topological clustering result for the 20,205 single-cells using 83 consensus TDGs for 10 cell types in human developmental pancreas organoids (s3–s6). The gene expression distribution of the three most influential genes (*GCG*, *ISL1*, and *SST*) revealed by VKO analysis.

**Additional file 10 Table S4.** 83 consensus TDGs for s3–s6 human developmental pancreas organoids and their GO enrichment analysis.

**Additional file 11 Table S5.** Virtual knockout genes and performances of pancreas developmental topology reconstructions by running topological clustering 100 times (score = accuracy + ARI).

**Additional file 12 Table S6.** Datasets and performances of 3D structure reconstructions with highly variable genes selected by Seurat (score = accuracy + ARI).

## Data Availability

The proposed methods including feature gene selection, 3D reconstruction using stochastic-SOM clustering, and visualization are implemented in R and available at https://github.com/tmorikuicr/espresso. All the gene expression data used in this study are included in the package.

## Abbreviations

ARI: Adjusted Rand index
BMU: Best matching unit
CPM: Counts per million mapped reads
ES: Embryonic stem
FISH: Fluorescence in situ hybridization
FPKM: Fragments per kilobase of exon per million mapped reads
GEO: Gene Expression Omnibus
GGM: Graphical Gaussian model
GO: Gene Ontology
iPS: Induced pluripotent stem
MCMC: Markov chain Monte Carlo
PCA: Principal component analysis
PCW: Postconceptional week
SA: Simulated annealing
scRNA-seq: Single-cell RNA-sequencing
SDG: Spatial discriminator gene
SOM: Self-organizing map
TDG: Temporal discriminator gene
UMAP: Uniform manifold approximation and projection
UMI: Unique molecular identifier
VKO: Virtual knockout
