# Supplementary material for: eSPRESSO: topological clustering of single-cell transcriptomics data to reveal informative genes for spatio–temporal architectures of cells
Source: BMC Bioinformatics. 2023 Jun 15;24:252. doi: 10.1186/s12859-023-05355-4 (PMC10268514; doi:10.1186/s12859-023-05355-4)
Supplement: Supplementary file 1 — Additional file 1 Fig. S1. GO mapping of SDGs in Biological Process for mouse E7.0 gastrula embryo. The p-values determined on the basis of hypergeometric distribution statistics are shown on top of bars. Fig. S2. Topology graphs for 12 datasets used as input for eSPRESSO clustering in this study. Fig. S3. Gene expression gradients in reconstructed models by eSPRESSO (upper) and novoSpaRc (lower) for the top three important genes in VKO analysis of mouse developmental heart. Fig. S4. Gene expression gradients in reconstructed models by eSPRESSO (upper) and novoSpaRc (lower) for the top three important genes in VKO analysis of the human developmental pancreas. Fig. S5. GGM raw result by glasso for original 18 cell types at individual stages (left) and induced developmental model by reduced 10 cell types (right). Fig. S6. Venn diagrams representing consensus S(T)DGs identified by eSPRESSO and cluster biomarkers identified by Seurat, and enrichment plots of the S(T)DGs for the gene lists ranked according to the adjusted p-values obtained by the Wilcoxon rank sum test between one domain and the other domains. P.adj and NES denote adjusted p-value and normalized enrichment score, respectively. Fig. S7 Synthetic single-cell dataset created from a dyngen model with linear backbone of transcription factor module configuration, and a topology graph generated from the dataset. a Module network of linear backbone generated by dyngen. b Transcription factor and target gene regulatory network generated by dyngen. c UMAP of 1,000 cells of the synthetic dataset. Numbers in the legend are the cluster numbers identified by the Louvain method. d Topology graph generated from the synthetic dataset. Vertex labels indicate domain names corresponding to the cluster numbers in (c). Fig. S8. Topology graphs generated from synthetic single-cell datasets of predefined 14 backbones by dyngen. The dyngen parameters for generating each dataset are indicated by Seed, random seed; #TF, the number [file 12859_2023_5355_MOESM1_ESM.pdf]

eSPRESSO: Topological clustering of single-cell transcriptomics data to reveal informative genes for spatio-temporal architectures of cells

Supplementary Material

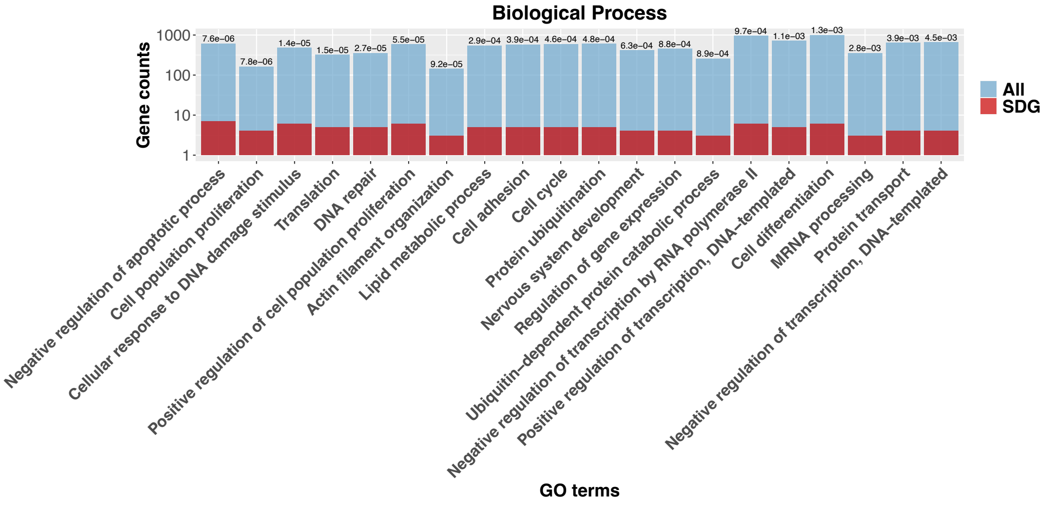

Fig. S1 GO mapping of SDGs in Biological Process for mouse E7.0 gastrula embryo. The p-values determined on the basis of hypergeometric distribution statistics are shown on top of bars.

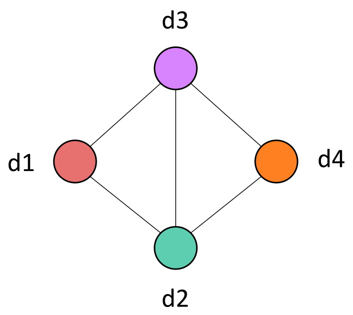

Mouse embryo (E7.0), 4 domains

d1: Anterior
d2: Lateral-distal
d3: Lateral-proximal
d4: Posterior

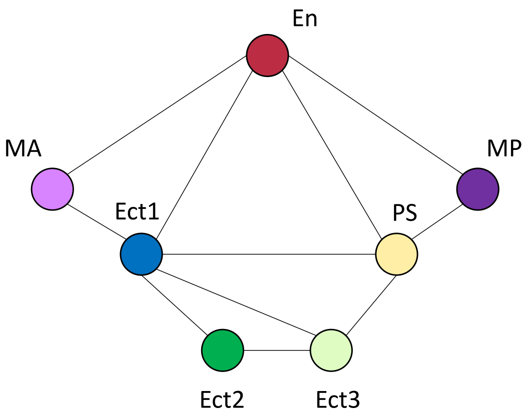

Mouse embryo (E7.5), 7 domains

Ect1-3: Ectoderm
PS: Primitive streak
MA: Anterior mesoderm
MP: Posterior mesoderm
En: Endoderm

Fig. S2 Topology graphs for 12 datasets used as input for eSPRESSO clustering in this study.

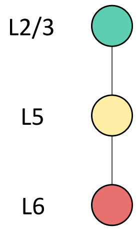

Mouse brain (ALM), 3 domains

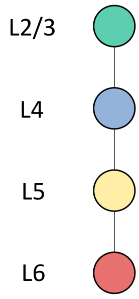

Mouse brain (VISp), 4 domains

Fig. S2 (continued).

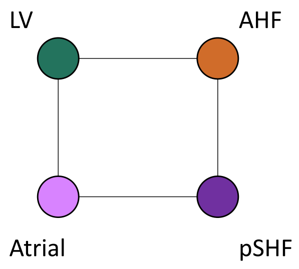

Mouse heart (E7.75)

AHF: Anterior heart field
LV: Left ventricle
pSHF: Posterior second heart field

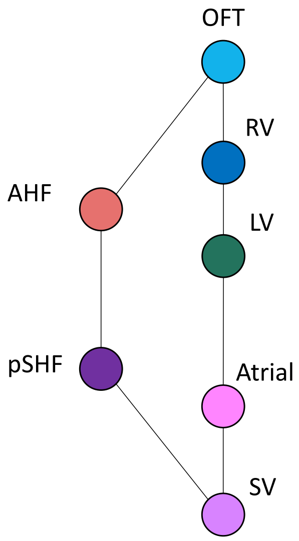

Mouse heart (E8.25 & E9.25)

AHF: Anterior heart field
LV: Left ventricle
pSHF: Posterior second heart field
OFT: Second heart field-derived outflow tract
RV: Right ventricle
SV: Sinus venosus

Fig. S2 (continued).

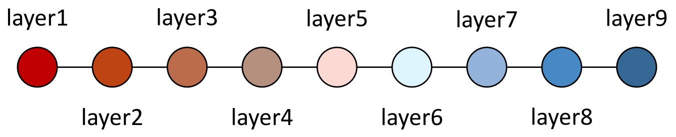

Mouse liver

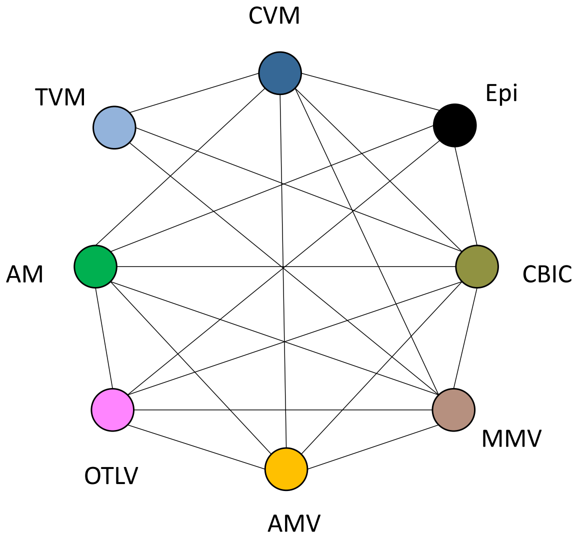

Human heart (4.5-5 PCW & 6.5 PCW) (dense version)

CVM: Compact ventricular myocardium
TVM: Trabecular ventricular myocardium
AM: Atrial myocardium
OTLV: Outflow tract / large vessels

AMV: Atrioventricular mesenchyme and valves
MMV: Mediastinal mesenchyme and vessels
CBIC: Cavities with blood and immune cells
Epi: Epicardium

Fig. S2 (continued).

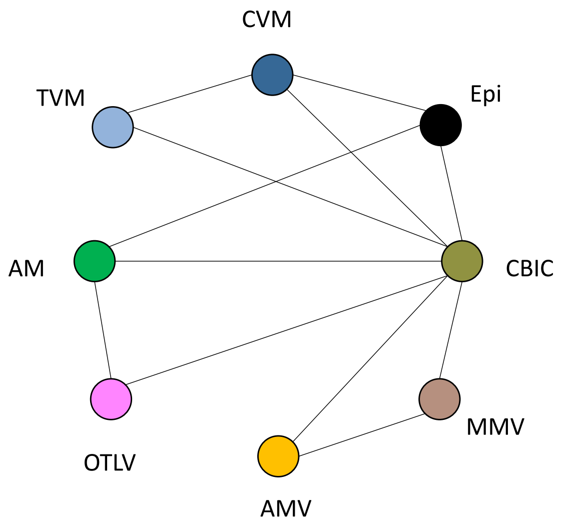

Human heart (4.5-5 PCW & 6.5 PCW) (sparse version)

CVM: Compact ventricular myocardium
TVM: Trabecular ventricular myocardium
AM: Atrial myocardium
OTLV: Outflow tract / large vessels

AMV: Atrioventricular mesenchyme and valves
MMV: Mediastinal mesenchyme and vessels
CBIC: Cavities with blood and immune cells
Epi: Epicardium

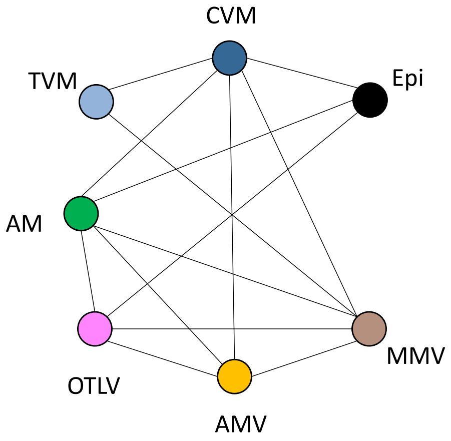

Human heart (9 PCW) (dense version)

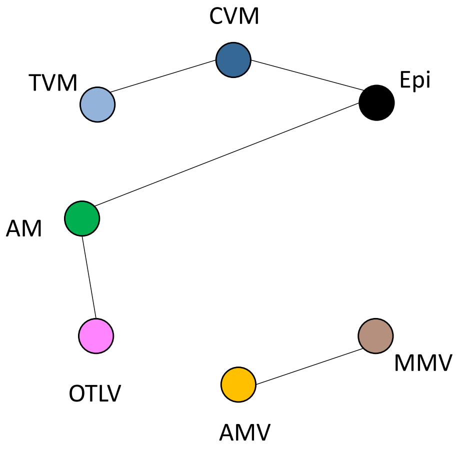

Human heart (9 PCW) (sparse version)

CVM: Compact ventricular myocardium
TVM: Trabecular ventricular myocardium
AM: Atrial myocardium
OTLV: Outflow tract / large vessels

AMV: Atrioventricular mesenchyme and valves
MMV: Mediastinal mesenchyme and vessels
Epi: Epicardium

Fig. S2 (continued).

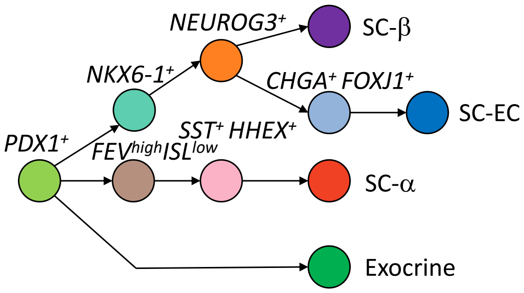

Human pancreas organoid (s3-s6) developmental model derived from Fig.5 in Veres *et al.* Nature (2019) **569**:368-373.

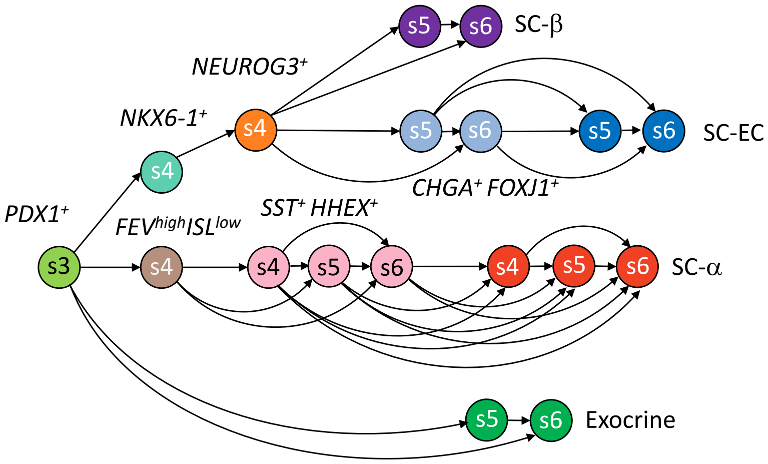

Topology used in eSPRESSO for human pancreas organoid (s3-s6) developmental model derived from Fig.5 in Veres *et al.* Nature (2019) **569**:368-373.

Fig. S2 (continued).

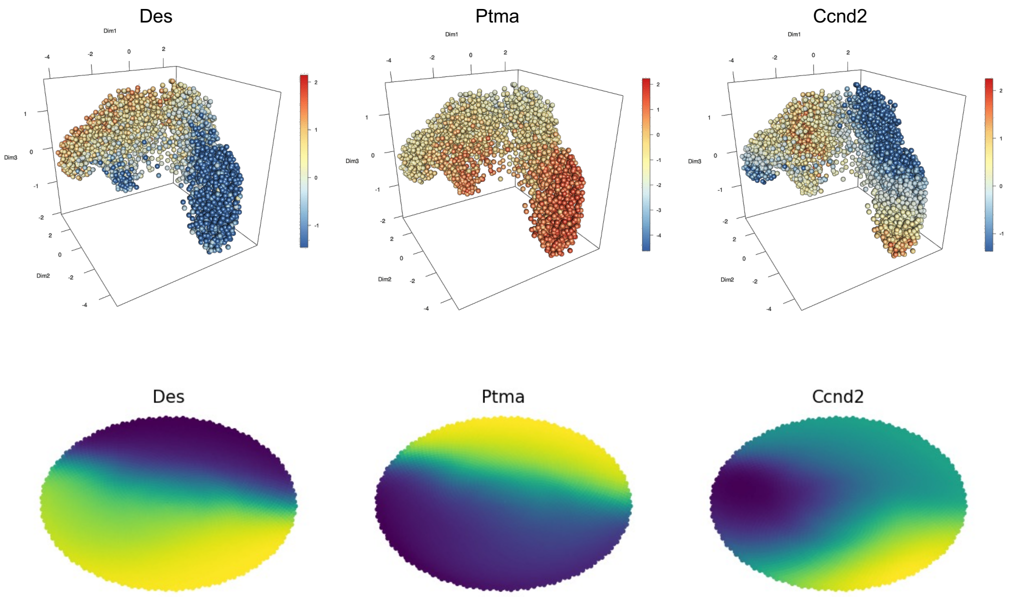

Fig. S3 Gene expression gradients in reconstructed models by eSPRESSO (upper) and novoSpaRc (lower) for the top three important genes in VKO analysis of mouse developmental heart.

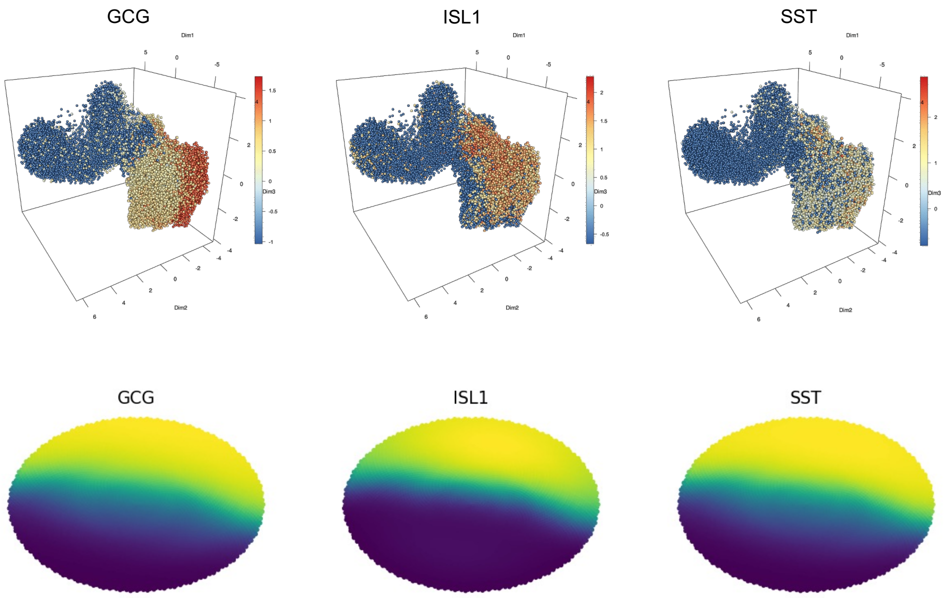

Fig. S4 Gene expression gradients in reconstructed models by eSPRESSO (upper) and novoSpaRc (lower) for the top three important genes in VKO analysis of the human developmental pancreas.

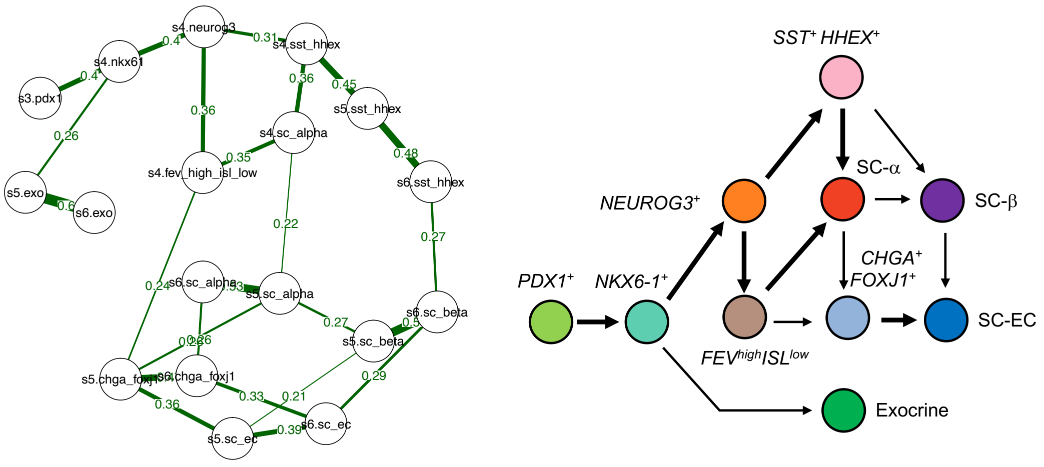

Fig. S5 GGM raw result by glasso for original 18 cell types at individual stages (left) and induced developmental model by reduced 10 cell types (right).

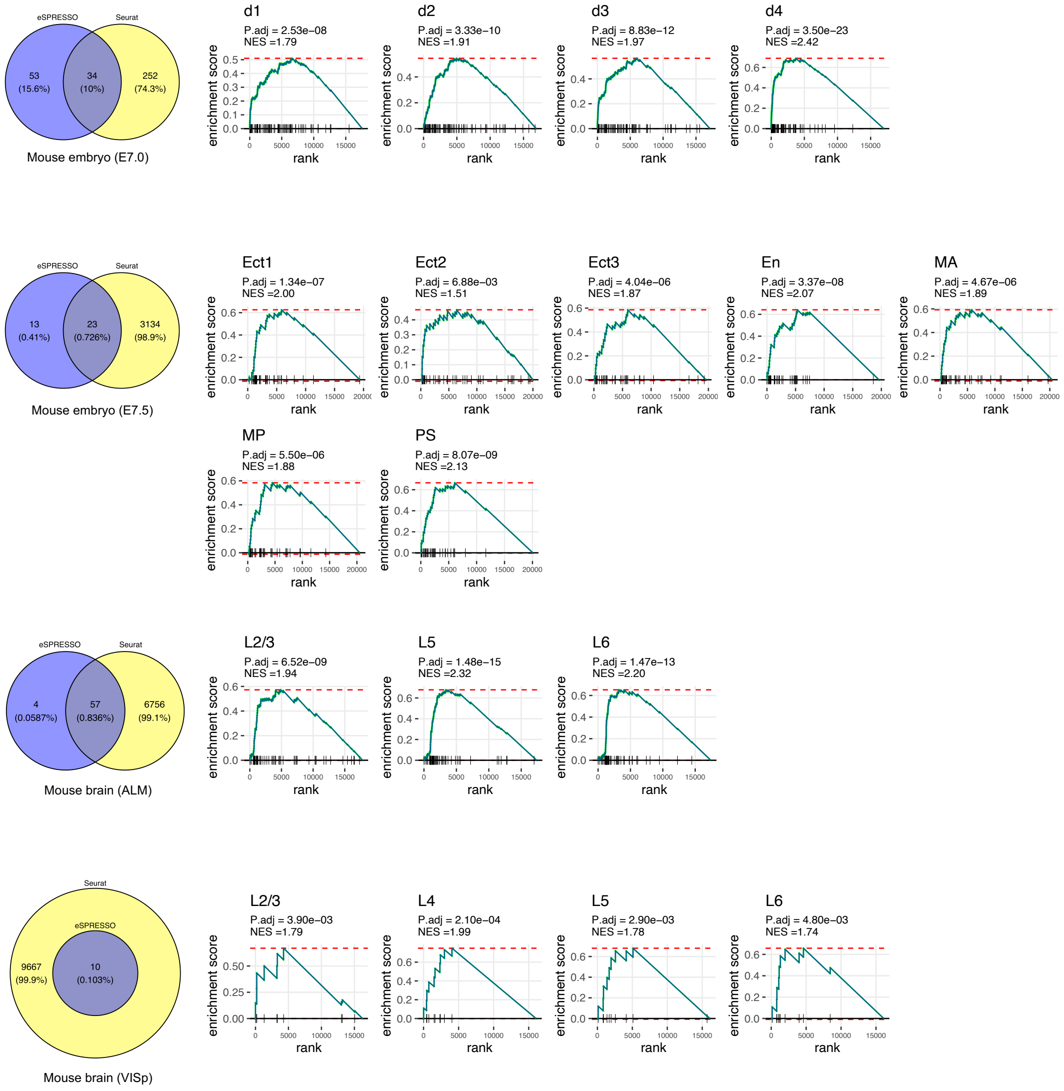

Fig. S6 Venn diagrams representing consensus S(T)DGs identified by eSPRESSO and cluster biomarkers identified by Seurat, and enrichment plots of the S(T)DGs for the gene lists ranked according to the adjusted p-values obtained by the Wilcoxon rank sum test between one domain and the other domains. P.adj and NES denote adjusted p-value and normalized enrichment score, respectively.

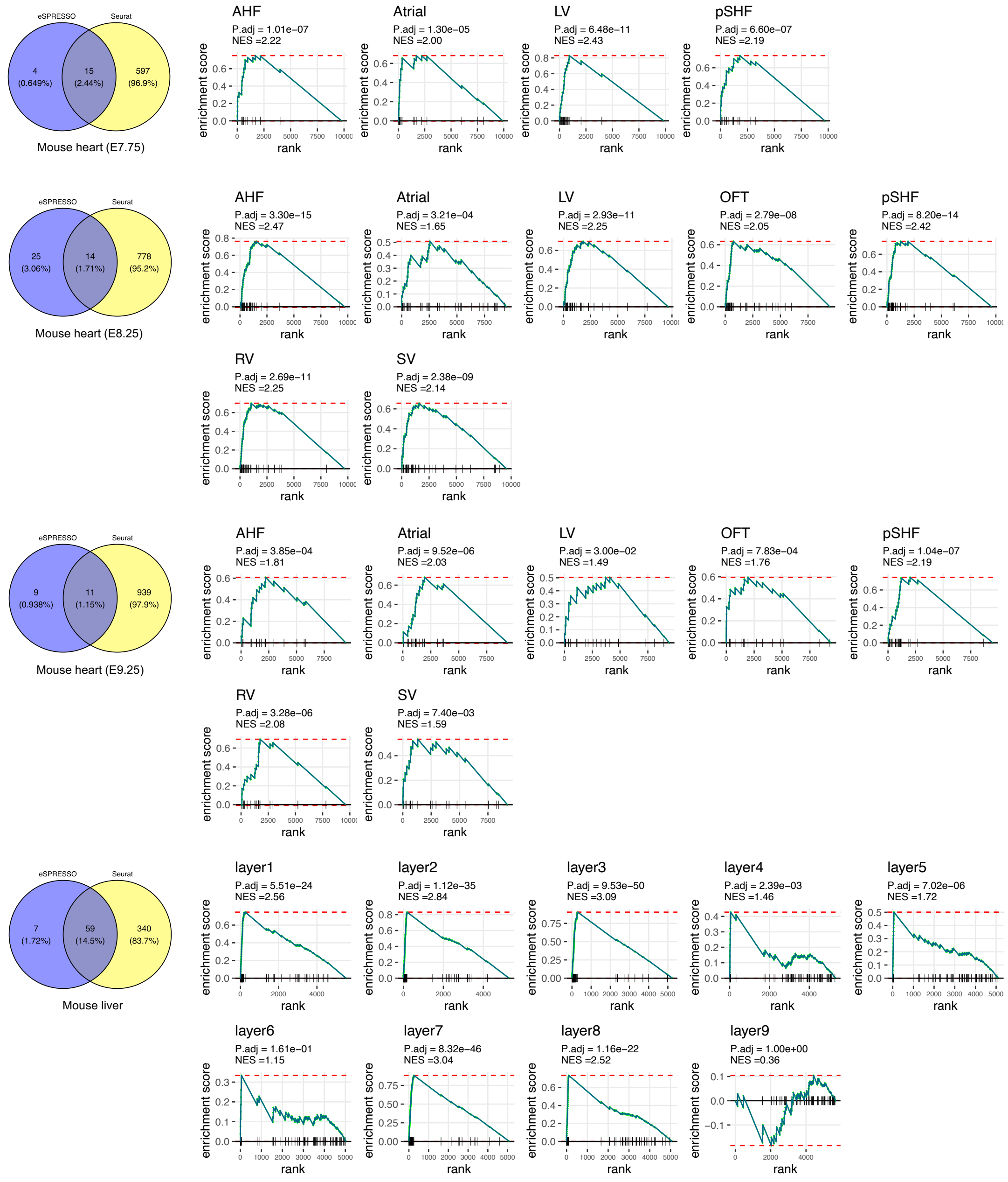

Fig. S6 (continued).

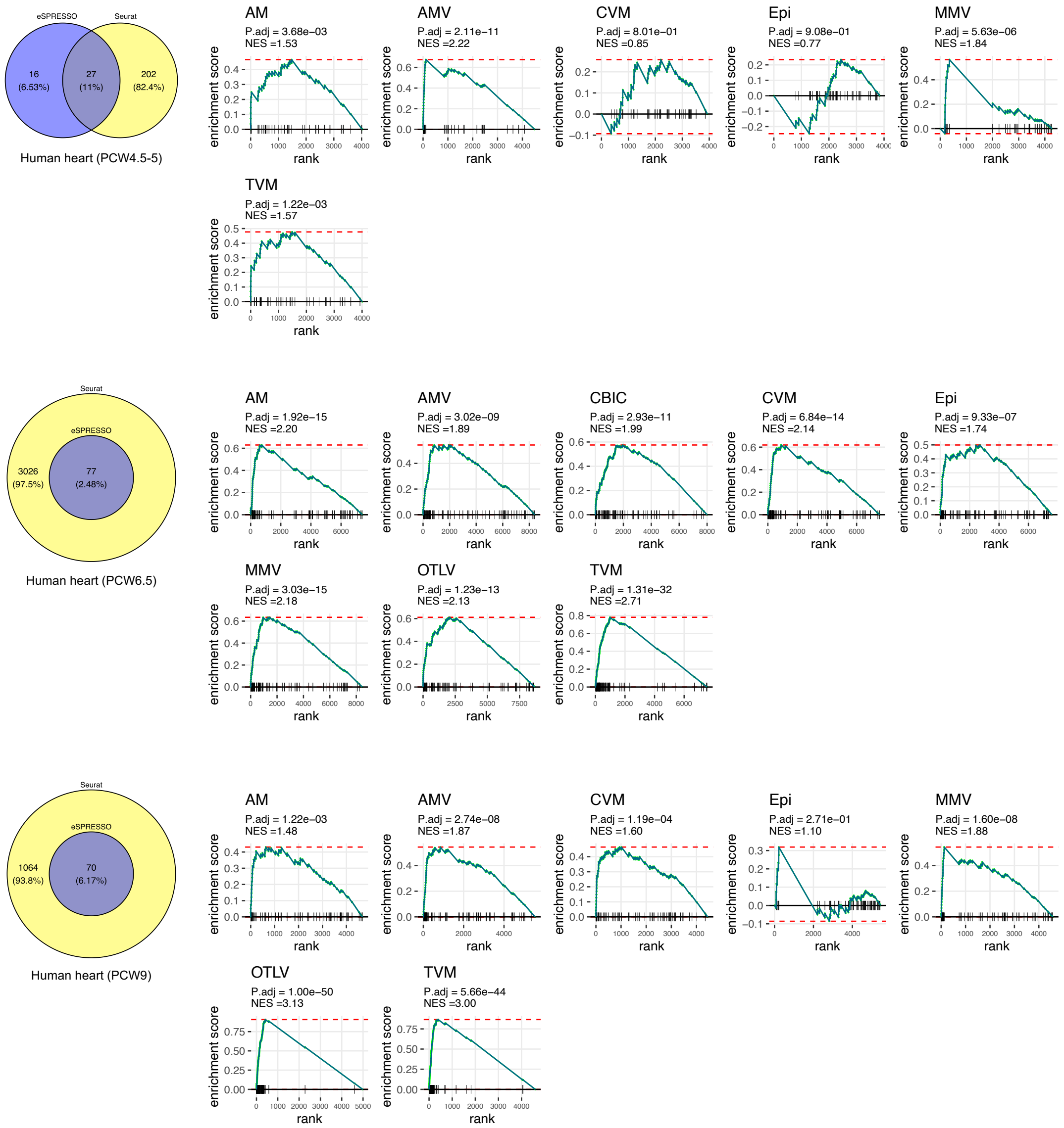

Fig. S6 (continued).

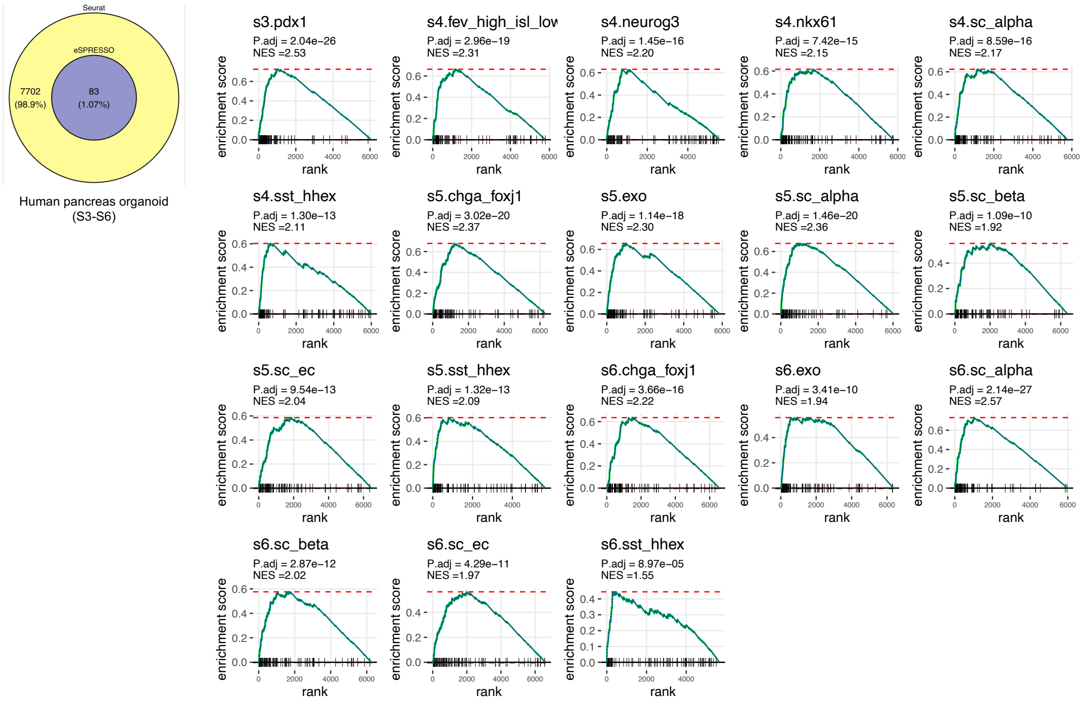

Fig. S6 (continued).

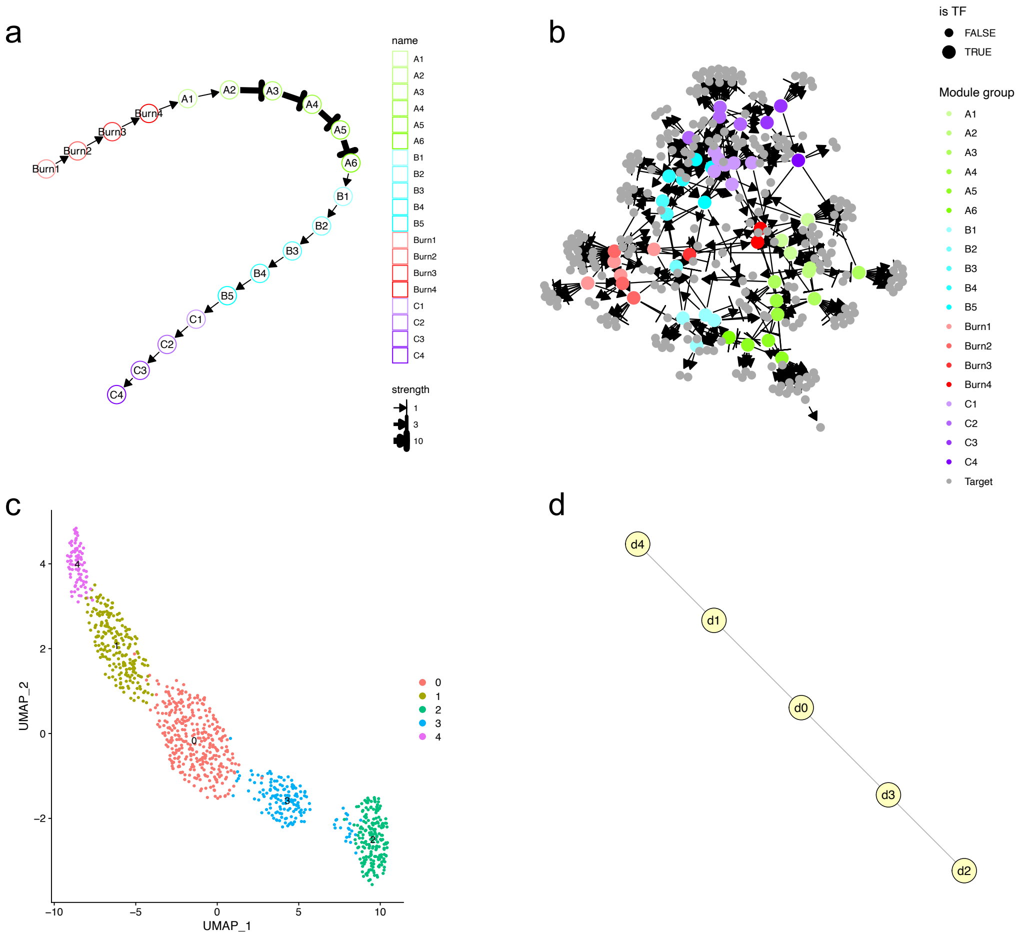

Fig. S7 Synthetic single-cell dataset created from a dyngen model with *linear* backbone of transcription factor module configuration, and a topology graph generated from the dataset. **a** Module network of *linear* backbone generated by dyngen. **b** Transcription factor and target gene regulatory network generated by dyngen. **c** UMAP of 1,000 cells of the synthetic dataset. Numbers in the legend are the cluster numbers identified by the Louvain method. **d** Topology graph generated from the synthetic dataset. Vertex labels indicate domain names corresponding to the cluster numbers in (**c**).

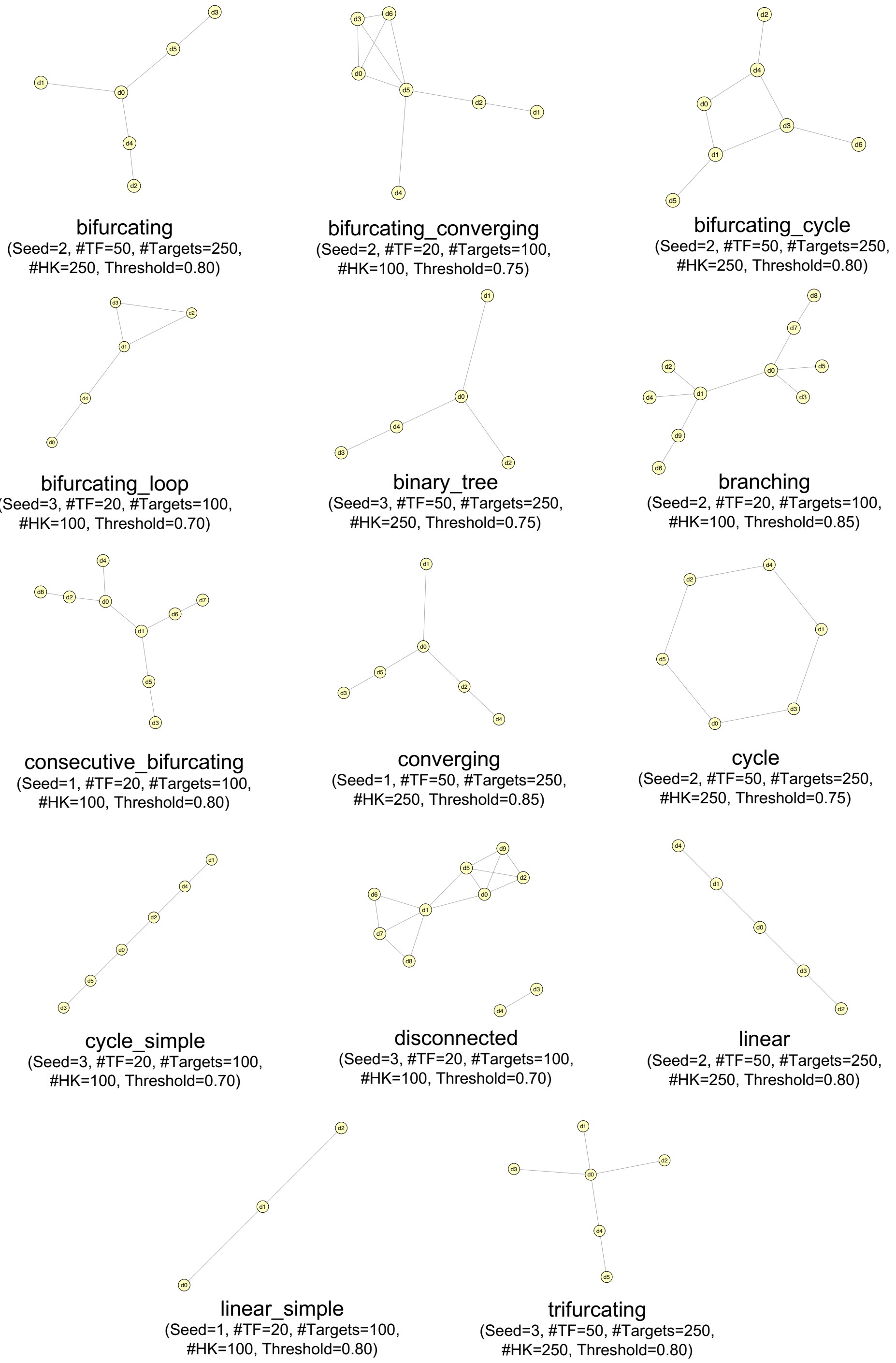

Fig. S8 Topology graphs generated from synthetic single-cell datasets of predefined 14 backbones by dyngen. The dyngen parameters for generating each dataset are indicated by Seed, random seed; #TF, the number of transcription factors; #Targets, the number of target genes; and #HK, the number of housekeeping genes. Thresholds indicate the cut-off values for generating topology graphs from the correlation networks of identified domains.

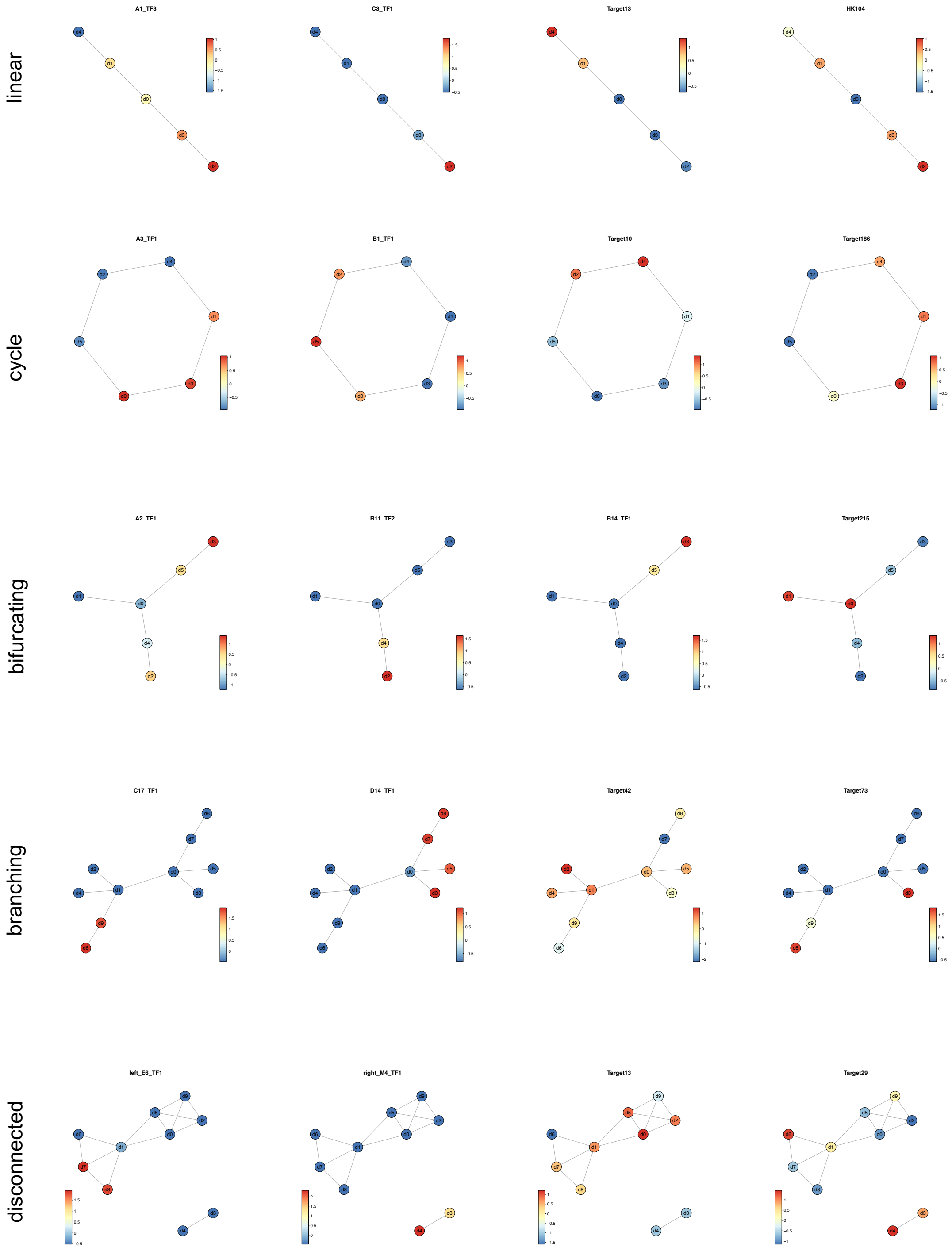

Fig. S9 Expression gradients of SDGs identified by eSPRESSO on topology graphs generated from the synthetic datasets. Vertex colors indicate gene expression levels (Z-score) of the cluster centroids.

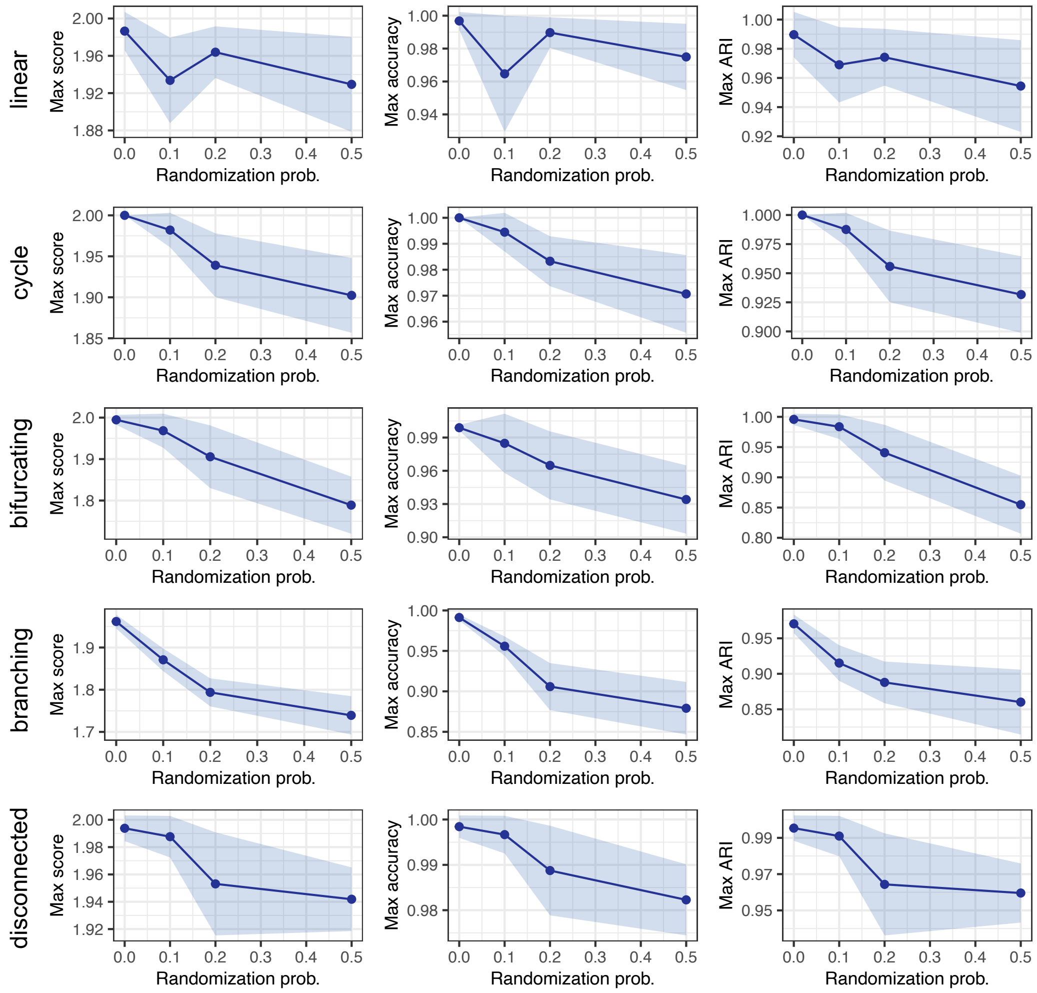

Fig. S10 Impact of misspecification of input topology. Each plot shows the mean values of maximum scores (= accuracy + ARI), maximum accuracies, and maximum ARIs, respectively, and 95% confidence intervals for ten experiments with increasing randomization probability.

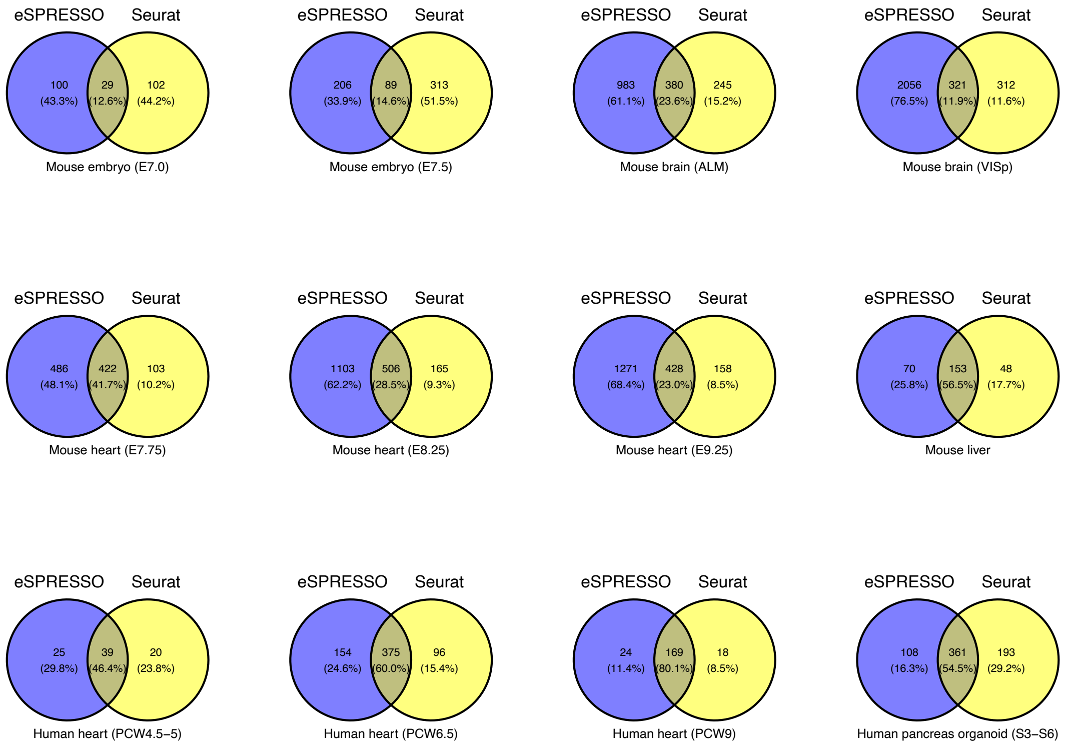

Fig. S11 Venn diagrams representing input genes to Boruta selected on the basis of the frequency of nonzero expression in all cells as well as the standard deviation (eSPRESSO) and highly variable genes by Seurat (Seurat).
